# Global prevalence of norovirus gastroenteritis after emergence of the GII.4 Sydney 2012 variant: a systematic review and meta-analysis

**DOI:** 10.3389/fpubh.2024.1373322

**Published:** 2024-06-27

**Authors:** Pan Zhang, Cai Hao, Xie Di, Xue Chuizhao, Li Jinsong, Zheng Guisen, Liu Hui, Duan Zhaojun

**Affiliations:** ^1^College of Public Health, Gansu University of Traditional Chinese Medicine, Lanzhou, Gansu, China; ^2^National Key Laboratory of Intelligent Tracking and Forecasting for Infection Diseases, NHC Key Laboratory of Medical Virology and Viral Diseases, National Institute for Viral Disease Control and Prevention, Chinese Center for Disease Control and Prevention, Beijing, China; ^3^Chengdu Kanghua Biological Products Co., Ltd., Chengdu, China; ^4^National Institute of Parasitic Diseases, Chinese Center for Disease Control and Prevention (Chinese Center for Tropical Diseases Research), NHC Key Laboratory of Parasite and Vector Biology, WHO Collaborating Center for Tropical Diseases, National Center for International Research on Tropical Diseases, Shanghai, China

**Keywords:** norovirus, gastroenteritis, prevalence, meta-analysis, genotype

## Abstract

**Introduction:**

Norovirus is widely recognized as a leading cause of both sporadic cases and outbreaks of acute gastroenteritis (AGE) across all age groups. The GII.4 Sydney 2012 variant has consistently prevailed since 2012, distinguishing itself from other variants that typically circulate for a period of 2–4 years.

**Objective:**

This review aims to systematically summarize the prevalence of norovirus gastroenteritis following emergence of the GII.4 Sydney 2012 variant.

**Methods:**

Data were collected from PubMed, Embase, Web of Science, and Cochrane databases spanning the period between January 2012 and August 2022. A meta-analysis was conducted to investigate the global prevalence and distribution patterns of norovirus gastroenteritis from 2012 to 2022.

**Results:**

The global pooled prevalence of norovirus gastroenteritis was determined to be 19.04% (16.66–21.42%) based on a comprehensive analysis of 70 studies, which included a total of 85,798 sporadic cases with acute gastroenteritis and identified 15,089 positive cases for norovirus. The prevalence rate is higher in winter than other seasons, and there are great differences among countries and age groups. The pooled attack rate of norovirus infection is estimated to be 36.89% (95% CI, 36.24–37.55%), based on a sample of 6,992 individuals who tested positive for norovirus out of a total population of 17,958 individuals exposed during outbreak events.

**Conclusion:**

The global prevalence of norovirus gastroenteritis is always high, necessitating an increased emphasis on prevention and control strategies with vaccine development for this infectious disease, particularly among the children under 5 years old and the geriatric population (individuals over 60 years old).

## 1 Introduction

Norovirus (NoV) is a non-enveloped, single-stranded RNA virus belonging to the Caliciviridae family, with a genome length of approximately 7.5 kb and a diameter ranging from 26 to 40 nm (1). Genogroups are further classified into capsid genogroup (genotypes) or P-genogroup (genotypes), which are determined based on the divergence of the VP1 capsid (ORF2) amino acid sequence or nucleotide diversity in the RNA-dependent RNA polymerase (RdRp; ORF1) region, respectively. Based on the capsid genogroup, NoV have been classified into ten genogroups (GI ~ GXI). Among these genogroups, GI, GII, GIV, GVIII, and GIX have been identified in humans (2). GII genogroup has a significantly higher prevalence compared to others (3, 4). Presently, there are 48 distinct capsid genotypes and 60 unique P-genotypes. Additionally, a dual-nomenclature system has been proposed to integrate both the RdRp and VP1 sequences, in response to the possibility of recombination events occurring between ORF1 and ORF2 (2). NoV is widely recognized as a leading cause of both sporadic cases and outbreaks of acute gastroenteritis (GE) across all age groups. The populations most susceptible to norovirus gastroenteritis (NoVGE) may encompass infants, the older adult, and individuals with compromised immune systems (5). According to Nadim et al., the global prevalence of NoV in community cases of GE was reported as 24%, while the prevalence in outbreaks was reported as 38% (6). NoV caused more than 130,000 people globally in 2019, with over 43,000 deaths occurring among children under the age of five and an additional 54,000 deaths among individuals aged 70 or above. NoVGE resulted in substantial direct health system costs amounting to $4.2 billion (95% UI: $3.2–5.7 billion) annually, along with societal costs reaching $60.3 billion (95% UI: $44.4–83.4 billion) (7, 8).

NoV is highly contagious and can spread rapidly in closed settings such as hospitals, schools, and cruise ships (9). NoV outbreaks occur year-round, with a higher incidence during colder seasons. Furthermore, NoV is responsible for a significant proportion of foodborne illnesses worldwide, with contaminated food and water serving as the main routes of transmission (10). The majority of these outbreaks predominantly manifest in educational institutions such as schools and kindergartens in China, primarily through person-to-person transmission, foodborne transmission, and waterborne transmission, and multiple ways of co-transmission (11). Prior to 2012, there existed five distinct NoV GII.4 variants that caused a worldwide pandemic and underwent replacement every three to four years. However, since the emergence of the GII.4 Sydney 2012 variant, it has gained extensive global circulation. To enhance our understanding of the distribution of NoVGE during the GII.4 Sydney 2012 variant era, we conducted a comprehensive meta-analysis spanning from 2012 to 2022 with the aim of assessing the current global pooled prevalence of this disease.

## 2 Materials and methods

### 2.1 Study area and period

This study analyzed the global prevalence and distribution characters of NoVGE from 2012 to 2022. The distribution characteristics included: (1) the geographical distribution: the prevalence distribution in different countries, in the northern and southern hemispheres, in developed and developing countries, as well as in China and its neighboring countries; (2) temporal distribution: prevalence distribution in different years, in months, in seasons adjusted for local temperature variations, and in cold season and warm seasons; (3) population distribution: prevalence variation in human populations with regards to age groups and gender stratification; (4) examination of the composition of NoV genotypes among sporadic cases; (5) Based on the relevant literature data obtained through the strategy for retrieving literature, this study analyzed the size of outbreaks, their attack rate, as well as the composition of NoV genotypes. We excluded papers which did not have an English abstract, did not show the number of patients with acute GE or patients positive for NoV, or percentages that could be used for calculating prevalence.

### 2.2 Literature source

#### 2.2.1 Search strategy

We searched the PubMed, Embase, Web of Science, and Cochrane databases between January 2012 and August 2022. The following search terms were used as a text word in each database:“Norovirus” or “Norwalk,” “caliciviruse” and “Morbidity,” “positive rate,” “detection rate,” “attack rate” or “prevalence”, limited GE cases or Diarrhea cases. The literature was screened by reading the title, and after eliminating irrelevant literature, study eligibility was further assessed by reading the abstract and full text.

#### 2.2.2 Quality control, criteria for inclusion, and exclusion

The literature underwent independent screening, selection, and cross-validation by two researchers. In the event of any disagreements, a third researcher was consulted for resolution. Initially, two reviewers independently selected articles that fulfilled the study requirements based on their titles and abstracts. During the initial screening process, articles meeting the following conditions were excluded: (1) articles published in languages other than English; (2) The GE cases were not caused by NoV but by other caliciviruses; (3) The subjects were not humans; (4) data derived from specific patient groups, such as transplant recipients and immunocompromised patients; (5) The pathogens were detected by antigen assays such as ELISA and immune-assays, not by PCR-based diagnostics methods. (6) The subjects were infected with NoV by human intervention rather than natural infection, such as volunteer challenge studies. (7) The articles were opinion articles and editorial articles, such as review articles, case reports, posters, and conference abstracts.

We conducted a comprehensive analysis of the entire texts of the remaining articles to identify those that met our research criteria. During this phase, we excluded the articles with the following characteristics: (1) papers that did not report the number of patients with acute GE or patients testing positive for NoV, or provide percentages that could be used to calculate pooled prevalence; (2) papers with a study period less than 12 months for sporadic cases surveillance; (3) studies with a sample size of less than 30 participants; and (4) If multiple studies present the same data, priority was given to the study with the highest level of comprehensiveness. In addition, we have excluded studies that monitor sporadic cases conducted by sub-municipal units and those focusing on an isolated outbreak event.

#### 2.2.3 Data extraction

The following information was extracted from each eligible article: the last name of the first author, year of publication, study location, specimen Research Topic time (year / month), disease type, number of cases, age ranges and gender of participants, number of NoV-positive cases, genogroup and genotype of NoV. The classification of a country as developed or developing was determined based on the World Bank’s economic development criteria outlined in this website (12). The extracted data were imported into a pre-designed Excel spreadsheet (Microsoft Corporation) (13).

### 2.3 Statistical analysis

The statistical analysis in this study was conducted using R (4.1.2) software, the shapiro.test function from the stats package was employed for assessing the normality of the pooled original prevalence of NoVGE. Use the original incidence rate or perform a logarithmic transformation to make it follow a normal distribution, and perform meta-analysis on it using the metaprop function included in the metapackage. The forested.meta function was utilized to generate the forest plot, while the funnel function was used for drawing the funnel plot. The Egger test and sensitivity analysis are performed by metabios function. Additionally, sensitivity analysis forest plots were created using the forest function. A significance test level of *p* < 0.05 was employed in the meta-analysis, while heterogeneity levels were categorized as high when I2 exceeded 50% and very high when I2 surpassed 75%. The comparison of rates and component ratios was conducted using the chi-square test, with statistical significance defined as *p* < 0.05.

## 3 Results

### 3.1 Study characteristics

**Figure 1:**
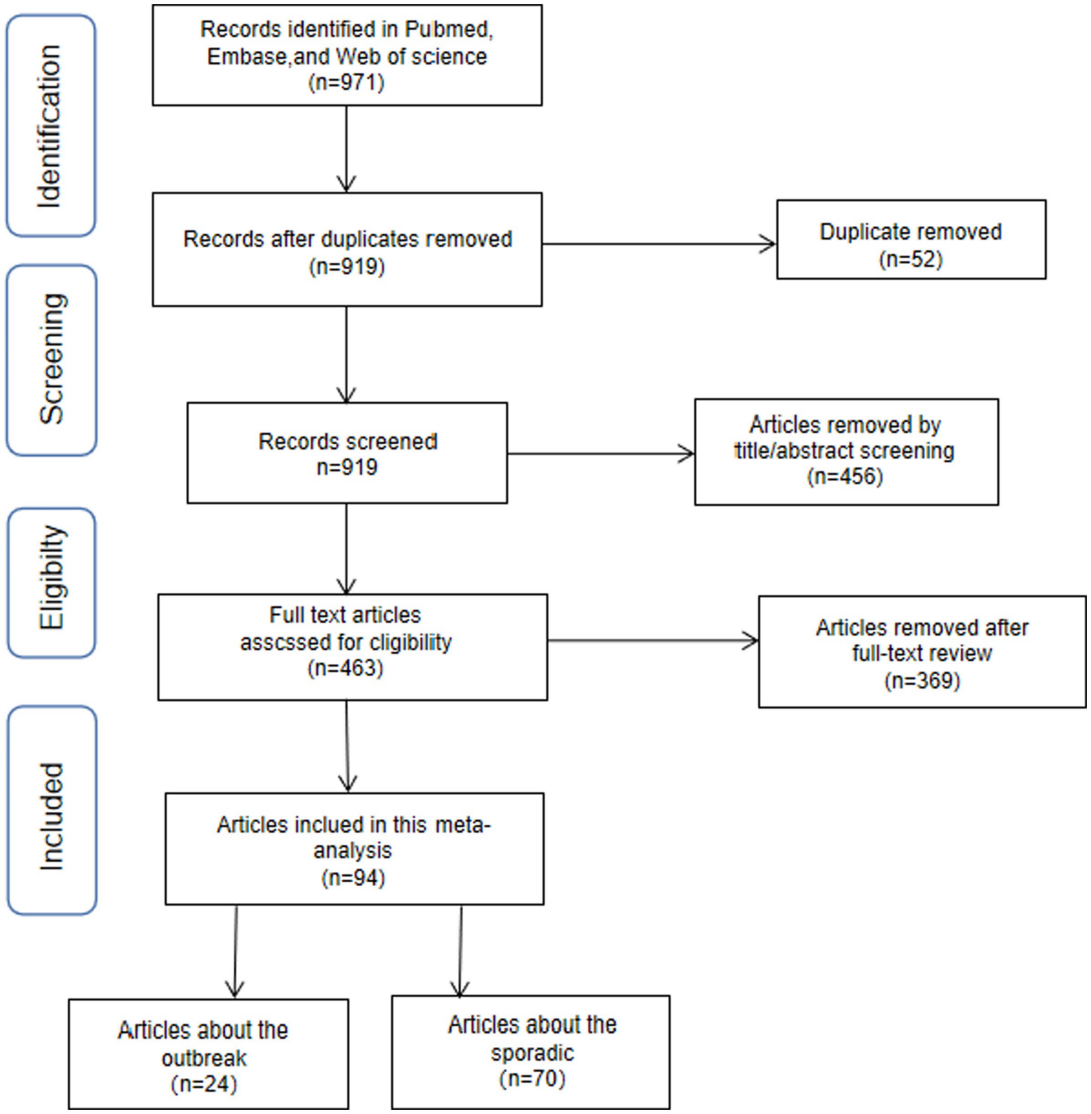
Flowchart presenting the steps of literature search and selection Distribution of the sporadic NoV cases.

A total of 971 articles were initially identified in the search, with 883 retrieved from PubMed, 52 from Web of Science, 5 from Cochrane, and 31 from Embase. Fifty-two duplicate articles were excluded first, and 456 additional articles were excluded after review of titles and abstracts. The remaining 463 articles underwent a thorough assessment through full text reading. Among these, a total of 283 articles lacking data or figures or tables necessary for calculating the pooled prevalence of NoVGE were excluded. Furthermore, after careful evaluation, another set of 86 articles was excluded due to incomplete or unusable data. Ultimately, a total of 94 papers were considered to have good quality. A comprehensive overview illustrating the selection process is presented in Figure 1 and Table 1.

**Table 1:** Characteristics of studies included in the systematic review and meta-analysis.

Author	Publication year	Location	Disease type	Number of Cases/exposed in outbreak	Number of positive cases
Saho Honjo (14)	2022	Japan	Sporadic	457	182
Jing Ai (15)	2022	Jiangsu, China	Outbreak	396,105	10,306
Yaoska Reyes (16)	2022	Nicaragua	Sporadic	1,353	229
Pattara Khamrin (17)	2022	Thailand	Sporadic	889	154
Noemi Navarro-Lleó (18)	2022	Spain	Sporadic	4,950	471
Gillian A. M. Tarr (19)	2021	Albert, Canada	Sporadic	3,319	898
Mahadeb Lo (20)	2021	East India	Sporadic	2,812	170
Gédéon Prince Manouana (21)	2021	Gabon	Sporadic	177	26
Takako Utsumi (22)	2021	East Java, Indonesia	Sporadic	966	119
Sylvia Kahwage Sarmento (23)	2021	Brazil	Sporadic	1,546	496
Alberto Ignacio (24)	2021	Amazon	Sporadic	485	184
Mahadeb Lo (20)	2021	East India	Sporadic	2,812	170
Ignacio Parrón (25)	2021	Catalonia, Spain	Outbreak	4,631	1,201
Chengxi Sun (26)	2021	Shandong, China	Outbreak	/	/
Scott Grytdal (27)	2020	U.S.A	Sporadic	1,603	103
Meghana P. Parikh (28)	2020	Tennessee, USA	Outbreak	3,273	755
Lei Ji (29)	2020	Huzhou, China	Sporadic	551	100
Daniel Hungerford (30)	2020	Blantyre, Malawi	Sporadic	683	83
Weiwei Shen (31)	2020	Taizhou, China	Sporadic	1,464	139
Baisong Li (32)	2020	Chongqing, China	Outbreak	/	1,637
Liping Chen (33)	2020	Huzhou, China	Outbreak	450	199
Miao Jin (34)	2020	China	Outbreak	/	/
Juan I. Degiuseppe (35)	2020	Argentina	Outbreak	/	189
Yi He (36)	2020	Shanghai, China	Outbreak	/	/
Ignacio Parrón (37)	2020	Catalonia, Spain	Outbreak	451	175
Marco André Loureiro Tonini (38)	2020	Brazil	Sporadic	272	65
Belinda L. Lartey (39)	2020	Ghana	Sporadic	1,337	485
Weiwei Shen (31)	2019	Taizhou, China	Sporadic	1,464	139
Mohammad Enayet Hossain (40)	2019	Bangladesh	Sporadic	613	109
A. Gelaw (41)	2019	Northwest Ethiopia	Sporadic	450	60
Hera Nirwati (42)	2019	Indonesia	Sporadic	406	75
Liang Xue (43)	2019	China	Sporadic	217	43
Evandro Leite Rodrigues Bitencurt (44)	2019	Brazil	Sporadic	240	38
Julianne R. Brown (45)	2019	London, UK	Outbreak	182	/
Hui-ying Li (46)	2019	Hohhot, China	Sporadic	1863	450
Lijuan Lu (47)	2019	Shanghai China	Sporadic	1,433	220
Takumi Motoya (48)	2019	Ibaraki, Japan	Outbreak	4,588	2,681
Shilu Mathew (49)	2019	Qatar	Sporadic	600	177
Zhiyong Gao (50)	2019	Beijing, China	Outbreak	762	661
Kanti Pabbaraju (51)	2019	Canada	Outbreak; Sporadic	/; 755	/; 94
Vivaldie Mikounou Louya (52)	2019	Congo	Sporadic	545	148
Betina Hebbelstrup Jensen (53)	2019	Denmark	Sporadic	688	103
Jianguang Fu (54)	2019	Jiangsu, China	Outbreak	/	3,951
Kgomotso Makhaola (55)	2018	Botswana	Sporadic	484	45
Jiankang Han (56)	2018	Huzhou, China	Sporadic	1,001	204
Mahsa Farsi (57)	2018	Tehran, Iran	Sporadic	210	36
E. Pagani (58)	2018	Northern Italy	Sporadic	702	162
Young Eun Kim (59)	2018	Seoul, South Korea	Sporadic; Outbreak	1,659; 2,414	271; 518
Massimiliano Bergalloa (60)	2018	Northern Italy	Sporadic	192	78
Victoria Kiseleva (61)	2018	Russia	Sporadic	429	49
Lanzheng Liu (62)	2018	Jinan, China	Outbreak	414	238
Caoyi Xue (63)	2018	Shanghai, China	Sporadic	5,927	1,363
Rosa Joosten (64)	2017	Netherlands	Outbreak	/	/
Leigh M. Howard (65)	2017	Lusaka, Zambia	Sporadic	454	52
Victor S. Santos Ricardo Q (66)	2017	Brazil	Sporadic	1,432	280
Mehme Özkan Timukan (67)	2017	Erzurum, Turkey	Sporadic	427	86
Giovanni Maurizio Giammanco (68)	2017	Italy	Sporadic	2,603	316
T. N. Hoa-Tran (69)	2017	Vietnam	Sporadic	350	99
S. Niendorf (70)	2017	Germany	Outbreak	240	175
Sonam Wangchuk (71)	2017	Bhutan	Sporadic	623	147
Jennifer L. Cannon (72)	2017	United States	Outbreak	/	/
Nafissatou Ouédraogo (73)	2016	Ouagadougou, Burkina Faso	Sporadic	263	55
Seyed Dawood Mousavi Nasab (74)	2016	Tehran, Iran	Sporadic	170	15
Zufan Sisay (75)	2016	Ethiopia	Sporadic	213	54
Peng Zhang (76)	2016	Huzhou, China	Sporadic	746	196
Leesa D (77).	2016	Australia	Outbreak	/	/
Casey L. (78)	2016	Bolivia	Sporadic	201	69
Yaqing He (79)	2016	Shenzhen, China	Outbreak	/	/
Makoto Kumazaki (80)	2016	Japan	Outbreak	1,497	1,050
Nada M Melhem (81)	2016	Lebanon	Sporadic	739	83
Hee Soo Koo (82)	2016	Busan, South Korea	Sporadic	2,174	49
C. F. Manso (83)	2015	Spain	Sporadic	2,750	747
Sonia Etenna Lekana-Douki (84)	2015	Gabon	Sporadic	317	73
Xiaofang Wu (85)	2015	Huzhou, China	Sporadic	796	211
Ben A. Lopman (86)	2015	Ecuador	Sporadic	438	79
Dongmei Tan (87)	2015	Nanning, China	Sporadic	342	101
Zhiyong Gao (88)	2015	Beijing, China	Sporadic	3,832	263
Heejin Ham, M. S (89).	2015	Seoul, South Korea	Sporadic	1,685	302
Jiankang Han (85)	2015	Huzhou, China	Sporadic	809	193
Jae-Seok Kim (90)	2015	Korea	Sporadic	2,980	349
Makoto Kumazak (91)	2015	Japan	Outbreak	947	
Ji-Hyuk Park (90)	2015	Korea	Outbreak	/	/
Masaki Yoneda (92)	2014	Nara, Japan	Sporadic	274	32
Ainara Arana (93)	2014	Chipsqua, Spain	Sporadic	4,574	714
James Ayukepi Ayukekbong (94)	2014	Cameroon Linbei	Sporadic	2,458	100
João Rodrigo Mesquita (95)	2013	Global	Sporadic	373	83
Meng-Bin Tang (96)	2013	Taiwan, China	Sporadic	155	17
Carmen F. Manso (97)	2013	Southern Spain	Sporadic	2,643	747
Amy A. Saupe (98)	2013	U.S.A	Sporadic	1,060	127
Maria E. Hasing (99)	2013	Canada	Outbreak	/	/
Hyun Soo Kim (100)	2013	Korea	Sporadic	1718	254
Pascale Huynena (101)	2013	Burkina Faso	Sporadic	418	93
Nguyen V. Trang (102)	2012	Vietnam	Sporadic	501	180
Mei Zeng (103)	2012	China	Sporadic	4,440	1,148

### 3.2 Distribution of the sporadic norovirus cases

**Figure 2:**
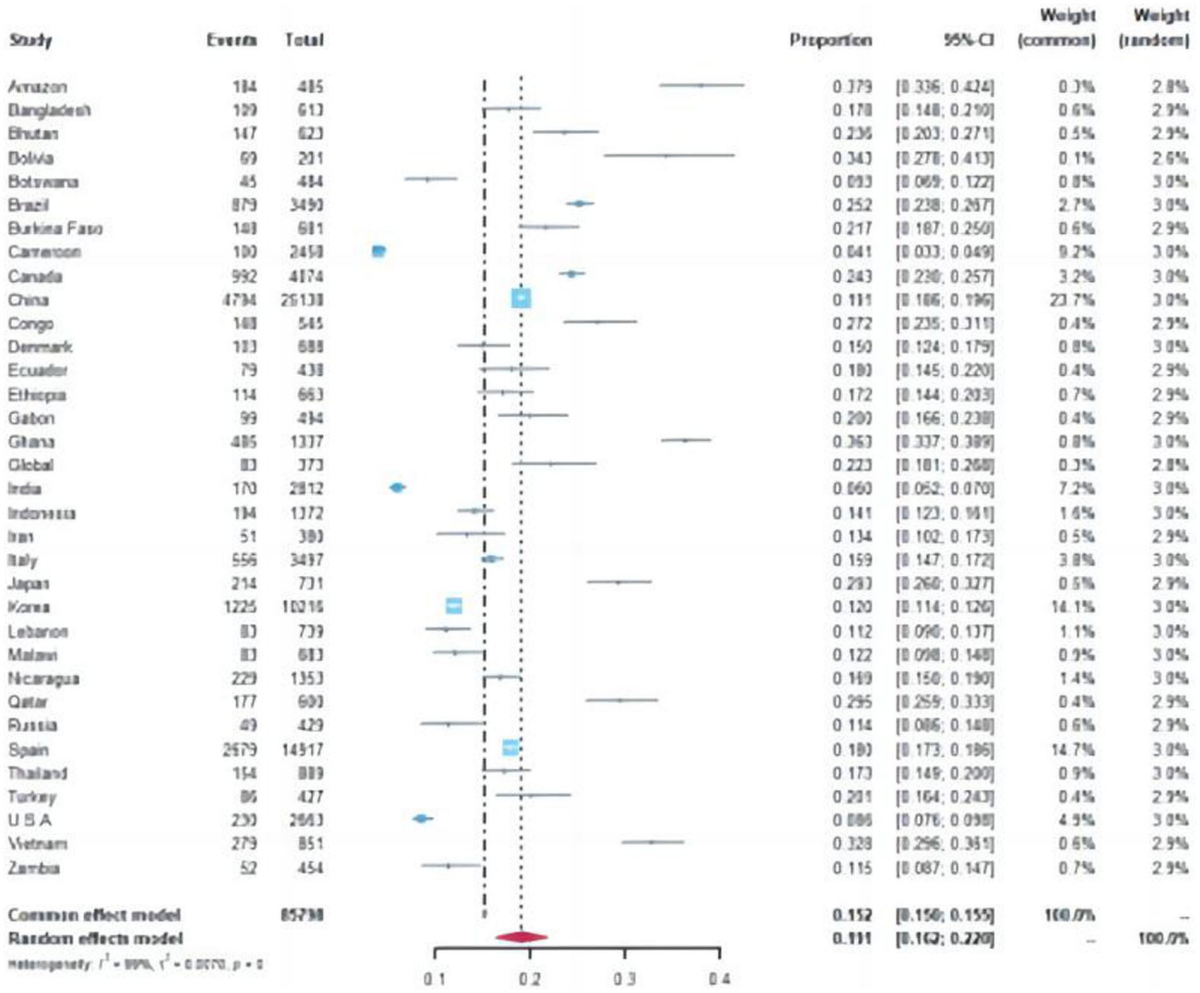
Forest plot of the pooled norovirus prevalence in the sporadic gastroenteritis cases from difference countries. CI, confidence interval.

**Figure 3:**
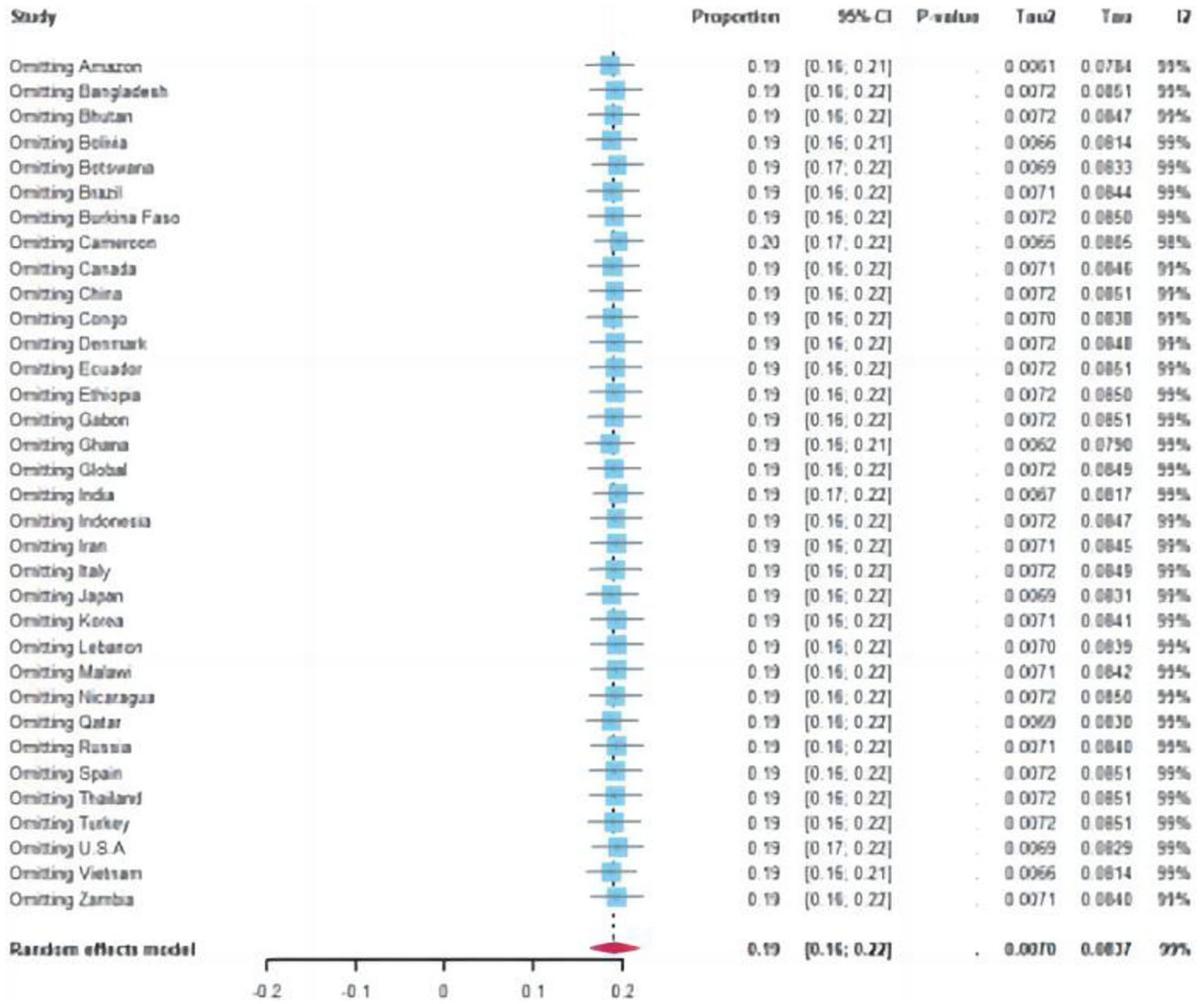
Funnel plot of the pooled norovirus prevalence in the sporadic gastroenteritis cases from difference countries. CI, confidence interval.

A total of 15,089 cases tested positive for NoV out of 85,798 sporadic cases with acute GE from 70 studies. The global average pooled prevalence of NoV in patients with acute GE was 19.04% (95%Cl:16.66–21.42%). Notably, the highest prevalence was observed in the Amazon region (37.94%), while Cameroon recorded the lowest prevalence at a mere 4.07%. Statistical analysis revealed an I^2^ value of 99% and a significance level of *p* < 0.001 (Figures 2, 3).

Specifically, the pooled prevalence in the Southern Hemisphere reached 20.02% (95% Cl: 19.32–20.72%), whereas it stood at 17.15% (95% Cl: 16.88–17.42%) in the Northern Hemisphere (Chi-square value = 41.6, *p* < 0–05) (Supplementary Figures S4.1, S4.2). Furthermore, developed countries demonstrated a lower positivity rate of 16.25% (95% Cl:15–88%-16–63%), whereas developing countries displayed a relatively higher positivity rate of 18–58% (95%C1:18–23%-18–93%) (Chi-square value = 55.34, *p* < 0.05).

**Figure 4:**
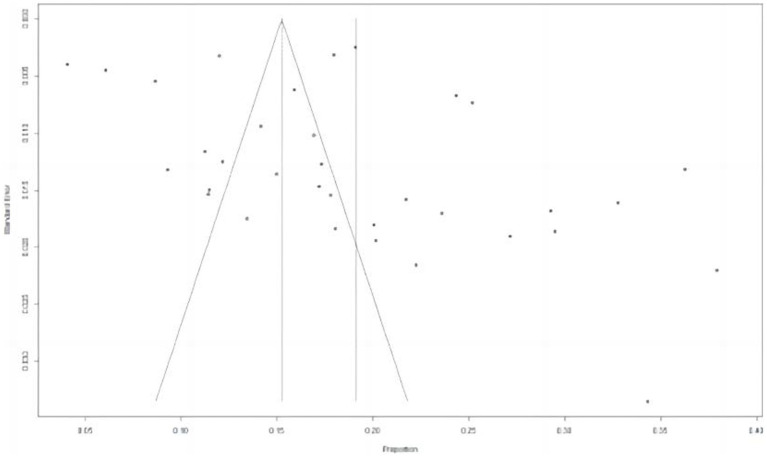
Funnel plot for assessment of publication bias.

Out of the 70 articles reviewed, 43 originated from China and its neighboring countries. The pooled prevalence of NoVGE patients, based on the 43 articles, was found to be 17.63% (95% Cl: 13.35–21.91%) (Supplementary Figures S1.1, S1.2). This prevalence was lower than that of China alone (19.07, 95% Cl: 18.59–19.56%) Additionally, Bangladesh and Thailand exhibited similar prevalence rates to China (Chi-square value = 0.44, *p* < 0.05; Chi-square value = 1.18, *p* < 0.05) (Supplementary Figures S2.1, S2.2). Japan and Vietnam demonstrated higher prevalence rates at 29.27% (95% Cl: 25.98–32.57%) and 32.78% (95% Cl: 29.63–35.94%), respectively. Conversely, Korea, Indonesia, and Russia showed relatively lower prevalence rates, with India reporting the lowest prevalence at 6.05% (95% Cl: 5–7). Furthermore, the prevalence of NoV in Zhejiang (19.12, 95% Cl: 18.63–19.61%) was significantly higher than that in Taiwan Province (10.97, 95% Cl: 6.05–15.89%) (Chi-square value = 4.84, *p* < 0.05) (Figure 4).

A total of 20 studies provided information on sample Research Topic, with contributions from China (5), Korea (3), Indonesia (2), the U.S.A. (2), and one each from Japan, the Amazon, Botswana, Brazil, India, Italy, and Thailand. Additionally, a separate study conducted simultaneous monitoring in multiple countries. The value of *I^2^* indicated a high level of heterogeneity at 98%, with *p* < 0.05 (Supplementary Figure S11.1). The average pooled prevalence based on these papers was approximately 16.01% (95% Cl: 15.59–16.44%). Notably, in 2015 the highest positive rate observed was 23.21% (95% Cl: 21.79–24.64%), followed by 21.07% (95% Cl:19 0.55%-22 0.59%) in 2017.

A total of nine articles, including two from China, four from Korea, and one each from Amazon, Indonesia, Italy, and the USA were analyzed to provide data on the prevalence of NoV in patients with GE on a monthly basis. The analysis revealed a significant level of heterogeneity (I^2^ = 98%, *p* < 0.05) (Supplementary Figures S5.1, S5.2). The overall prevalence of NoV was determined to be 16.52% (95% Cl: 12.49–20.54%). To account for seasonal differences between the northern and southern hemispheres, adjustments were made for their respective seasons as described above. Following these adjustments, it was found that December had the highest prevalence of NoV at 26.31% (95% Cl: 24.15–28.46%), followed by January at 23.44% (95% Cl: 21.46%-25–42%) and November at 23.32% (95% Cl: 21–18%-25–47%). In contrast, June exhibited the lowest prevalence of NoV at only 6–83% (95% Cl: 5 0.43%-8 0.23%).

Based on the aforementioned nine articles, an additional two papers were included in this analysis, revealing a prevalence of 16.25% (95% Cl: 8.84–23.66%), with a high level of heterogeneity (I^2^ = 100%, *p* < 0.05) (Supplementary Figures S6.1, S6.2). It is worth noting that during the winter months (December–February in the Northern Hemisphere and June–August in the Southern Hemisphere), a significantly higher prevalence of NoV was observed at 22.98% (95% Cl: 21.82–24.14%). In contrast, the summer months (June–August in the Northern Hemisphere and December–February in the Southern Hemisphere) exhibited a relatively lower prevalence of NoV at 7.05% (95% Cl: 6.23–7.79%).

A comprehensive analysis was conducted using data from the 11 aforementioned studies and an additional publication to examine the prevalence of NoV during warm seasons (April to September in the Northern Hemisphere, October to March in the Southern Hemisphere) and cold seasons (October to March in the Northern Hemisphere, April to September in the Southern Hemisphere). The statistical analysis revealed a high level of heterogeneity (I^2^ = 100%, *p* < 0.05) (Supplementary Figures S7.1, S7.2). The overall prevalence of NoV was determined to be 15.21% (95% Cl: 5.02–25.39%). Specifically, during warm seasons, the prevalence rate was 10.01% (95% Cl: 9.42–10.61%); however, this significantly increased to 20.41% (95% Cl: 19.66–21.15%) during cold seasons (*p* < 0.01).

### 3.3 Distribution of the norovirus outbreak

A total of 7,054 outbreak events were pooled from 24 studies across 10 countries: China (10), the United States (2), Japan (2), Canada (2), Spain (2), Argentina, Australia, Germany, South Korea, the Netherlands, and the United Kingdom. However, 15 articles were excluded due to insufficient information on the number of outbreaks or lack of information on cases or exposed individuals. Of the remaining nine articles, one reported a total of 10,563 individuals testing positive for NoV out of 396,797 exposed individuals with an attack rate of 2.66% (95%Cl: 2.55%–2.65%). Upon review of their raw data, it shows that the number of exposed people in a large collective unit is the same as the total number of people in the unit, particularly in settings such as universities where not all individuals may have been exposed. Therefore, exclusion is necessary. In the remaining 8 articles, a total of 6,992 individuals tested positive for NoV out of a population of 17,958 who were exposed to outbreaks, allowing for an estimated prevalence of NoV infection at 36.89% (95% CI: 36.24–37.55%). From 2012 to 2019, a total of 10 studies in these papers reported the time information of the NoV outbreak from [China (3), the United States (2), Spain (1), Canada (1), Netherlands (1), Australia (1), Japan (1)]. These data showed that there are 334, 1,389, 1,028, 776, 148, 183, 259, and 26 outbreaks events in each respective year from 2012 to 2019.

There were 6 studies [China (2), Spain (1), Canada (1), Netherlands (1), South Korea (1)] provide the months distribution of the outbreaks, showing that December had the highest number of outbreak events (155) followed by November (80), July had the lowest count of outbreak events (4). Analyzing seasonal distribution revealed that spring accounted for 621 norovirus outbreak events while summer recorded 122 events, Autumn and winter experienced relatively higher incidences with counts reaching 282 and 401 events, respectively, based on the data obtained from 12 studies [China (6), Spain (1), the United States (2), Canada (1), Italy (1), South Africa (1), and the Netherlands (1)].

### 3.4 Genotype of norovirus

A total of 34 articles from 17 countries were used to genotype analysis in sporadic cases, including Brazil (2 articles), Botswana (2 articles), Bhutan (1 articles), China (8 articles), Ethiopia (1 article), Ghana (1 article), Germany (1 article), Italy (2 articles), Indonesia (2 articles), Japan (2 articles), Korea (4 articles), Malawi (1 article), Nicaragua (1 article), Qatar (1 article), Spain (3 articles), Thailand (1 article) and Vietnam (1 article). These studies reported 4,944 patients infected with various strains including GI.2 to GI.19, GI. untyped, GII.1 to GII.17, and GII.20 to GII.22. Among them, the proportion of GII.4 was the highest, 1722 cases of GII.4 genotype were reported in 16 countries, accounting for 34.83% of the total. The second was GII.3, with 1,245 cases (25.18%) reported from 15 countries. The detection rate of GII.2 genotype was 8.54% in 15 countries, while GII.17, GII.6, GII.14, GII.5, and GII.1 was 4.25, 2.89, 2.60, 2.74, and 2.53%, respectively.

In 8 articles reporting norovirus genotype from1292 cases in China, GI accounted for 6.89% and GII accounted for 93.11%. The most genotype was GII.4 with 481 cases (37.23%). The second was GII.3, with 308 cases (23.84%). Then there were 124 cases of GII.17, accounting for 9.60%, and 117 cases of GII.2, accounting for 9.06%.

A total of 14 studies [China (5), Spain (2), Canada (1), Germany (1), Japan (1)] reported the genotype of norovirus in 5166 outbreak events, with GInorovirus accounting for 12.25% and GII norovirus accounting for 87.75%, with GII.4 being the most prevalent (41.54%%), followed by GII.2 (22.11%), GII.6 (5.50%), and GII.3 (5.34%).

### 3.5 Sensitivity analysis

Sensitivity analysis revealed no statistically significant differences, except for a few outlier studies that deviate from the overall estimate. Since all the studies fall within the 95% confidence interval, the pooled prevalence remains unaffected by individual study findings.

## 4 Discussion

Norovirus is a major human pathogen caused severe GE affecting people from vulnerable populations of all age (104). Over the past three decades, a multitude of genotypes, including antigenically distinct variants of GII.4 noroviruses, have emerged and circulated in the world. GII.4 result more than 50% of all norovirus infections globally (13). The last variant to emerge, Sydney_2012, has been circulating at high incidence worldwide for over a decade caused the most severe disease burden (105). In this study, the pooled prevalence and genetic diversity of NoV were analyzed from studies conducted on patients with GE and published between January 2011 and April 2012.

In this study, it showed that the significant positive rate of norovirus in patients with acute GE worldwide. The positive rate of norovirus in GE patients is 19.04% (95%Cl:16.66–21.42%), which is higher than others reported. Ahmed et al. estimated the pooled prevalences of NoV infection was 18% in all age (106) while Mohammad Farahmand estimated the pooled prevalence of NoV infection was 17.7%% (95Cl: 16.3–19.2%) among children with GE from 45 countries across the world from 2015 to 2020 (13). Manish M Patel estimated that the pooled positive rate is 12% in patients with severe GE cases among children <5 years of age and 12% (95% Cl:9–15%) of mild and moderate diarrhea cases among persons of all ages (107) (Supplementary Figures S9.1, S9.2). The positive rate of the norovirus in patients is influenced by multiple factors, including viral variations, the number of susceptible individuals, and the surveillance and monitoring capabilities of participating institutions. Consequently, inconsistencies in pooled positive rates may arise due to variations in literature sources selected based on different inclusion and exclusion criteria. The high positive rate showed that we need more considering targeted intervention.

Our analysis reveals significant disparities in positive rates across regions and countries, with the highest rate observed in the Amazon region reaching up to 37.9%(95%Cl:33.6–42.4%), while the lowest rate was recorded in Cameroon at only 4.1% (95%Cl:3.3–4.9%). These highest and lowest positive rate countries is same as other meta-analysis (108). Yingyin Liao et al. showed that there was no statistical difference in terms of norovirus prevalence among different national income level (109). But our data indicates that developing countries exhibit higher rates of positivity compared to developed nations. Furthermore, our combined positive rate surpasses the findings from the meta-analysis conducted by Gia Thanh Nguyen et al., which revealed a decline in prevalence from 18% (95% CI: 16–20%) for upper middle-income countries to 15% (95%Cl:13–18%) and 6% (95%Cl:3–10%) for lower middle- and low-income countries, respectively, in both developed and developing regions (110) (Supplementary Figures S3.1, S3.2). Similarly, there is a greater prevalence of norovirus in the Southern Hemisphere than in the Northern Hemisphere. These findings emphasize substantial regional variations in norovirus occurrence. Insufficient or limited data on sporadic cases and outbreaks have been collected from numerous countries; however, it is worth noting that unreported sporadic cases are prevalent in these regions, particularly Africa. Consequently, the global incidence of norovirus may be underestimated due to these factors. All above highlight the necessity for further investigation into underlying factors contributing to these differences in positivity rates such as implementation of pandemic control measures, availability of healthcare resources, and testing capabilities.

According to our analysis of population distribution characteristics, we found that infants below the age of two had a higher positivity rate of norovirus infection, with a recorded rate of 23.1% (95%Cl: 21.7–24.5%). Notably, adolescents aged between five and fourteen demonstrated peak infection rates at 27.8% (95%Cl: 23.8–32.1%), while individuals within the age range of five to twenty also displayed high levels at 27.6% (95%Cl: 24.2–31.3%). In contrast, older adult individuals over sixty exhibited significantly lower infection rates at 12.3% (95%Cl:10.9–13 0.8%). In previous studies, Gia Thanh Nguyen reported prevalence rates of 16% (95%Cl:14–18%) for children under five years and 17% (95%Cl:13–21%) for adults. Ahmed et al. highlighted the global prevalence of norovirus in different age groups, with rates of 18%(95% CI: 15–20%)for children under five years, 18% (95% CI: 13–24%) for individuals over the age of five, and 19% (95% CI: 17–21%)for mixed age groups. The highest positive rate was shown in adolescents (14.74%) and adults (14.74%), followed by children (14.17%) and older adults (14.05%) (110). The pooled attack rate of norovirus was 6.73% based on the data of 899 outbreak events, with a higher attack rate in North than in South China, while the highest attack rate was found among older adults (11.85%), followed by children (9.48%), adolescents (5.53%) and adults (4.55%) (111). Yingyin Liao’s study revealed a comparable prevalence of norovirus among children under 5 years old, individuals over 5 years old, and across all age groups (109). The variations in prevalence observed among the age groups in these studies can be attributed to disparities in data sources, which focus on different populations under surveillance across various regions, as well as the distinct categorization of age groups employed within the studies. Furthermore, a higher prevalence of males (19.3% [18–20%]) compared to females (18.% [17–19%]) was observed, which is consistent with the findings reported by Mohammad Farahmand et al., indicating that boys are more susceptible than girls to NoVGE (13). This gender disparity may be attributed to occupational or lifestyle factors, as males tend to have a higher likelihood of dining outside the home or having increased contact with larger social networks.

The years 2015–2017 witnessed a notable increase in norovirus positive rates, with values of 23.2 (21.8–24.6), 20.8 (19.6–22.0), and 21.1 (19.6–22.6) respectively. The positive rate during the cold season, at 20.4 (19.7–21.2), significantly exceeded that of the warm season, particularly during winter where it reached a notably higher rate of 23.0% (21.8–24.2%). Among all months, December and January exhibited the highest rates with values of 26.3 (24.2–28.5) and 23.4 (21.5–25.5), respectively. Changes in temperature had the greatest attributable risk for norovirus incidence in a long-term study of England and Wales (112). Our results also suggested that there is a positive correlation between rainfall and seasonality as previous study (113). Although our findings align with previous studies (113), it is imperative to acknowledge the inherent limitations in our data sources. To gain a comprehensive understanding of the global seasonality of norovirus disease and its influencing factors, further research is needed in monitoring regions with limited capacity over an extended period of time (Table 2).

**Table 2:** Analysis of the prevalence of norovirus in gastroenteritis.

Characteristics	Categories	Cases of studies	Pooled prevalence (%) (95% CI)	Heterogeneity test I^2^%, *p*-value	Differences between subgroups; χ^2^ test (*p*-value)
Age groups (1)	<2 year	827	23.1 (21.7–24.5)	97%, *p* < 0.01	148.9 (*p* < 0.01)
	2–5 years	94	13.5 (11.0–16.2)		
	5–14 years	130	27.8 (23.8–32.1)		
	14-60 years	782	19.0 (17.8–20.3)		
	>60 years	249	12.3 (10.9–13.8)		
	Total	2082	19.0 (14.3–23.7)		
Age groups (2)	<5 years	1,047	22.1 (21.0–23.3)	98%, *p* < 0.01	136.4 (*p* < 0.01)
	5-20 years	175	27.6 (24.2–31.3)		
	20-60 years	532	17.3 (16.0–18.7)		
	>60 years	249	12.3 (10.9–13.8)		
	Total	2003	19.7 (14.5–24.9)		
Sex	Male	1,337	19.3 (18.3–20.2)	0, *p* = 0.48	0.5 (*p* = 0.48)
	Female	787	18.7 (17.6–19.9)		
	Total	2,124	19.1 (18.3–19.8)		
Years	2012	518	16.4 (15.1–17.7)	98%, *p* < 0.01	374.8 (*p* < 0.01)
	2013	927	14.9 (14.0–15.8)		
	2014	492	15.2 (14.0–16.4)		
	2015	783	23.2 (21.8–24.6)		
	2016	929	20.8 (19.6–22.0)		
	2017	582	21.1 (19.6–22.6)		
	2018	222	9.8 (8.6–11.0)		
	2019	155	10.2 (8.7–11.7)		
	Total	4,608	16.2 (15.7–16.6)		
Month	N January, S July	413	23.4 (21.5–25.5)	98%, *p* < 0.01	668.5 (*p* < 0.01)
	N February, S August	270	18.8 (16.8–20.9)		
	N March, S September	326	22.0 (19.9–24.2)		
	N April, S October	215	17.1 (15.1–19.3)		
	N May, S November	159	12.2 (10.5–14.1)		
	N June, S December	85	6.8 (5.5–8.4)		
	N July, S January	96	6.9 (5.5–8.3)		
	N August, S February	133	9.1 (7.7–10.7)		
	N September, S March	166	12.2 (10.5–14.1)		
	N October, S April	296	20.3 (18.3–22.5)		
	N November, S May	348	23.3 (21.2–25.6)		
	N December, S June	422	26.3 (24.2–28.5)		
	Total	2,929	16.5 (12.5–20.5)		
Season	Spring	734	16.9 (15.8–18.0)	99%, *p* < 0.01	650.2 (*p* < 0.01)
	Summer	327	7.1 (6.3–7.8)		
	Autumn	847	18.1 (17.0–19.3)		
	Winter	1,160	23.0 (21.8–24.2)		
	Total	3,068	16.3 (8.8–23.7)		
Temperature	Warm season	981	10.0 (9.4–10.6)	100%, *p* < 0.01	459.4 (*p* < 0.01)
	Cold season	2,315	20.4 (19.7–21.2)		
	Total	3,296	15.2 (5.0–25.4)		
Counties	Amazon	184	37.9 (33.6–42.4)	99%, *p* < 0.01	2947.1 (*p* < 0.01)
	Bangladesh	109	17.8 (14.8–21.0)		
	Bhutan	147	23.6 (20.3–27.1)		
	Bolivia	69	34.3 (27.8–41.3)		
	Botswana	45	9.3 (6.9–12.2)		
	Brazil	879	25.2 (23.8–26.7)		
	Burkina Faso	148	21.7 (18.7–25.0)		
	Cameroon	100	4.1 (3.3–4.9)		
	Canada	992	24.3 (23.0–25.7)		
	China	4,794	19.1 (18.6–19.6)		
	Congo	148	27.2 (23.5–31.1)		
	Denmark	103	15.0 (12.4–17.9)		
	Ecuador	79	18.0 (14.5–22.0)		
	Ethiopia	114	17.2 (14.4–20.3)		
	Gabon	99	20.0 (16.6–23.8)		
	Ghana	485	36.3 (33.7–38.9)		
	Global	83	22.3 (18.1–26.8)		
	India	170	6.0 (5.2–7.0)		
	Indonesia	194	14.1 (12.3–16.1)		
	Iran	51	13.4 (10.2–17.3)		
	Italy	556	15.9 (14.7–17.2)		
	Japan	214	29.3 (26.0–32.7)		
	Korea	1,225	12.0 (11.4–12.6)		
	Lebanon	83	11.2 (9.0–13.7)		
	Malawi	83	12.2 (9.8–14.8)		
	Nicaragua	229	16.9 (15.0–19.0)		
	Qatar	177	29.5 (25.9–33.3)		
	Russia	49	11.4 (8.6–14.8)		
	Spain	2,679	18.0 (17.3–18.6)		
	Thailand	154	17.3 (14.9–20.0)		
	Turkey	86	20.1 (16.4–24.3)		
	U.S.A	230	8.6 (7.6–9.8)		
	Vietnam	279	32.8 (29.6–36.1)		
	Zambia	52	11.5 (8.7–14.7)		
	Total	15,089	19.0 (16.7–21.4)		
Regions	Developed country	6,048	16.3 (15.9–16.6)	99%, *p* < 0.01	79.9 (*p* < 0.01)
	Developing Countries	8,958	18.6 (18.2–18.9)		
	Total	15,006	17.4 (15.1–19.7)		
	The Southern Hemisphere	2,485	20.0 (19.3–20.7)	98%, *p* < 0.01	55.5 (*p* < 0.01)
	The Northern Hemisphere	12,521	17.1 (16.9–17.4)		
	Total	15,006	18.6 (15.6–21.3)		
	Chinese Mainland	4,777	19.1 (18.6–19.6)	83%, *p* = 0.02	10.5 (*p* < 0.01)
	Taiwan	17	11.0 (6.5–17.0)		
	Total	4,794	15.4 (7.5–23.4)		

N: The Northern Hemisphere; S: The Southern Hemisphere. Spring: In the Northern Hemisphere, the period from March to May is considered spring, while in the Southern Hemisphere it occurs from September to November. Summer: From June to August, the Northern Hemisphere experiences summer, whereas in the Southern Hemisphere it takes place between December and February. Autumn: The months of September to November mark autumn in the Northern Hemisphere, while in the Southern Hemisphere it falls within March to May. Winter is defined as the period from December to February in the Northern Hemisphere and from June to August in the Southern Hemisphere, according to seasonal divisions. The warm and cold seasons were determined based on the mean monthly temperature of each city included in this study. In the Northern Hemisphere, the warm season spanned from April to September, while in the Southern Hemisphere it extended from October to March; all other months were designated as the cold season. The pooled prevalence of each subgroup was calculated using the random effects model in R software.

Genogroup GII is the leading NoV genogroup in the world. The GII.4 NoV genotype have been dominating in the past thirty years, in our review, a great diversity of NoV genotypes were reported ranging from GII.1 to GII.20, except GII.18, with the predominance of GII.4 (31.19%) from all identified genotypes. This report is also in agreement with previous studies where an increased prevalence of non-GII.4 NoV had been reported in different parts of China and Africa as a single variant or as a recombinant type (108, 114). In our study, the prevailing genotypes of norovirus in sporadic cases were found to be GII.4 (31.19%), GII.3 (24.73%), GII.2 (8.58%), GII.17 (4.31%), and GII.6 (2.94%); whereas the predominant genotypes in outbreak cases were observed as follows: GII.441.54%%, followed by GII.2 (22.11%), GII.6 (5.50%), and GII.3 (5.34%). The period from 2012 to 2014 accounted for approximately 66.40%(2751/4143) of outbreaks events maybe resulted by the endemic of norovirus GII.4 which caused about 41.54% outbreak events. In the United States, a surveillance study conducted from 2012 to 2016 also identified disparities in the prevalence of dominant norovirus genotypes between sporadic cases and outbreaks of GE (28). In the winter of 2014/15 GII.P17-GII.17 norovirus became a major cause of GE outbreaks in China and Japan and have replaced the previously dominant GII.4 genotype Sydney 2012 variant in some areas in Asia, which had been predicted as a strain to end the GII.4 era (115). However, the detection rate of GII.17 appears to be relatively lower, consistent with the findings reported by Yingyin Liao (109). This observation may potentially be attributed to the limited duration and geographical scope of the epidemic caused by GII.17 (105).

Previous research has indicated that males may have a higher susceptibility to NoVGE compared to females (male-to-female odds ratio: 1.1; 95%Cl: 1.03–1.3; I^2^ = 45.3%). This suggests both sex hormones and notable endocrine and genetic differences between males and females early in life may contribute to an increased vulnerability to NoV infection (13). In our analysis, we did not find a statistically significant gender-based distribution, although there was a slightly higher overall prevalence in men compared to women. This observation could be attributed to the inclusion of data from the adult population in our dataset (Table 3).

**Table 3:** The genotype composition of the sporadic NoVGE.

Country	Total	GI	GII	GII0.2	GII0.3	GII0.4	GII0.6	GII0.17	Other GII Genotypes
Bhutan	88	0	88 (100%)	2 (2.27%)	43 (48.86%)	26 (29.55%)	3 (3.41%)	2 (2.27%)	12 (13.63%)
Botswana	61	0	61 (100%)	3 (4.92%)	23 (37.70%)	23 (37.70%)	0	0	12 (19.67%)
Brazil	227	14 (6.17%)	213 (93.83%)	8 (3.52%)	36 (15.86%)	149 (65.64%)	4 (1.76%)	0	30 (13.22%)
China	1,292	89 (6.89%)	1,203 (93.11%)	117 (9.06%)	308 (23.84%)	481 (37.23%)	44 (3.41%)	123 (9.52%)	219 (16.95%)
Ethiopia	60	1 (1.67%)	59 (98.33%)	4 (6.67%)	0	21 (35.00%)	12 (20.00%)	8 (13.03%)	15 (25.00%)
Germany	134	13 (9.70%)	121 (90.30%)	61 (45.52%)	3 (2.23%)	40 (29.85%)	5 (3.73%)	6 (4.48%)	19 (14.18%)
Ghana	159	18 (11.32%)	141 (88.68%)	13 (8.18%)	86 (54.09%)	7 (4.40%)	0	0	53 (33.3%)
Indonesia	144	7 (4.86%)	137 (95.14%)	12 (8.33%)	21 (14.58%)	59 (40.97%)	11 (7.64%)	8 (5.56%)	33 (22.92%)
Italy	362	6 (1.66%)	356 (98.34%)	25 (6.91%)	11 (3.03%)	248 (68.51%)	21 (5.80%)	34 (9.39%)	23 (6.35%)
Japan	154	0	154 (100%)	53 (34.42%)	10 (6.49%)	70 (45.45%)	5 (3.25%)	12 (7.79%)	4 (2.60%)
Korea	1,225	100 (8.16%)	1,125 (91.84%)	78 (6.37%)	500 (40.82%)	201 (16.41%)	19 (1.55%)	1 (0.08%)	426 (34.78%)
Malawi	42	0	42 (100%)	2 (4.76%)	6 (14.29%)	20 (47.62%)	2 (4.76%)	1 (2.38%)	11 (26.19%)
Nicaragua	114	41 (35.96%)	73 (64.04%)	0	0	44 (38.60%)	1 (0.88%)	8 (7.02%)	61 (53.51%)
Qatar	177	2 (1.13%)	175 (98.87%)	24 (13.56%)	28 (15.82%)	110 (62.15%)	3 (1.69%)	5 (2.82%)	7 (3.95%)
Spain	498	27 (5.42%)	471 (94.58%)	13 (2.61%)	85 (17.07%)	173 (34.74%)	8 (1.61%)	1 (0.20%)	218 (43.78%)
Thailand	131	1 (0.76%)	130 (99.24%)	7 (5.34%)	66 (50.38%)	0	1 (0.76%)	0	57 (43.51%)
Vietnam	76	0	76 (100%)	0	19 (25.00%)	50 (65.79%)	4 (5.26%)	1 (1.32%)	2 (2.63%)
Total	4,944	319 (6.45%)	4,625 (93.55%)	422 (8.54%)	1,245 (25.18%)	1722 (34.83%)	143 (2.89%)	210 (4.25%)	1,202 (24.31%)

Efforts are warranted to advance the development of effective vaccines and control programs aimed at mitigating the burden of this substantial disease. Due to the considerable and rapid variability of norovirus, the coexistence of multiple dominant genotypes in the same time and space, the low cross-protection between genotypes, and the high incidence and severity outcomes among infants, older adult individuals, and patients with underlying diseases, we suggest that polyvalent vaccines containing multiple genotype-specific antigen epitopes as proposed by other researchers should be adopted and recommended for the above-mentioned population and tailored for the aforementioned high-risk populations. Several norovirus vaccine candidates have shown limited progress in clinical trials. However, Vaxart Pharmaceutical Inc. has developed an oral norovirus vaccine candidate utilizing recombinant adenovirus-based vectors carrying genes encoding norovirus VP1s to express antigens locally in the epithelial cells within the intestine of vaccine recipients, thereby inducing mucosal immunity (116, 117). This candidate vaccine has recently demonstrated a statistically significant reduction in infection rate, a non-statistically significant reduction in NoVGE, and a substantial reduction in viral shedding during challenge studies (118). These findings suggest that oral administration holds promise for the successful development of an effective norovirus vaccine.

## 5 Limitations

There are several limitations associated with this study. Firstly, there are publication bias which influence the results in our research findings. The majority of these publications have predominantly originated from developed regions and areas where English serves as the primary language, which may introduce a potential source of bias. Secondly, we collected as much published data as possible to mitigate the potential impact of inadequate sample size on our findings. Notably, the actual subgroup sample size significantly exceeded the calculated sample size, thereby enhancing the robustness and reliability of our results (S12). However, it is important to acknowledge that due to the diverse range of data sources utilized and inherent biases in available literature and data sources, there are certain limitations in achieving consistency across variables between studies, which may have some influence on our outcomes. In summary, the presence of heterogeneity in the literatures is apparent and should be taken into account when interpreting the findings of this review. Although our systematic approach and stringent inclusion criteria likely mitigated heterogeneity, biases inherent in the original studies, variations in study design and population, as well as publication bias cannot be completely eliminated.

## 6 Conclusion

Our findings reveal a high prevalence of NoVGE, yet significant data gaps exist in various regions, particularly in developing countries, indicating the absence of systematic surveillance for NoVGE in these areas. To reduce the incidence and diseases burden of NoVGE, it is crucial to enhance global standardized monitoring and collaboration, as well as develop cost-effective vaccines.

## Data availability statement

The original contributions presented in the study are included in the article/Supplementary material, further inquiries can be directed to the corresponding authors.

## Author contributions

PZ: Writing – original draft, Writing – review & editing. CH: Writing – original draft, Writing – review & editing. XD: Writing – original draft. XC: Methodology, Formal analysis, Writing – original draft. LJ: Data curation, Methodology, Supervision, Writing – review & editing. ZG: Supervision, Writing – review & editing. LH: Supervision, Writing – review & editing. DZ: Project administration, Supervision, Writing – review & editing.
