# Supplementary material for: Global prevalence of norovirus gastroenteritis after emergence of the GII.4 Sydney 2012 variant: a systematic review and meta-analysis
Source: Front Public Health. 2024 Jun 27;12:1373322. doi: 10.3389/fpubh.2024.1373322 (PMC11236571; doi:10.3389/fpubh.2024.1373322)
Supplement: Supplementary file 1 [file Data_Sheet_1.PDF]

S 1.1：Forest Plot Of China And Neighboring Countries

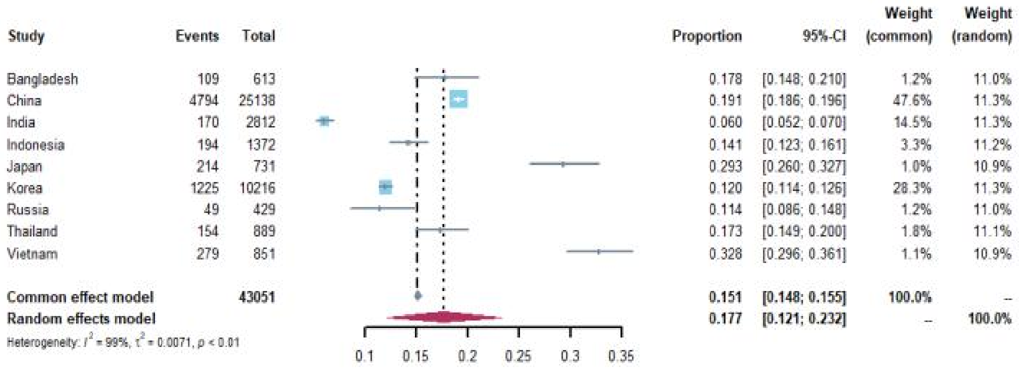

S 1.2：Sensitivity Analysis Chart Of China And Neighboring Countries

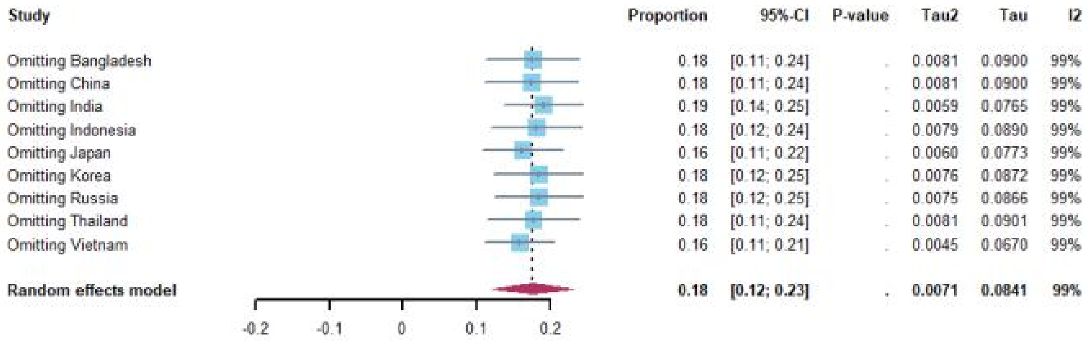

S 1.3：Funnel Plot Of China And Neighboring Countries

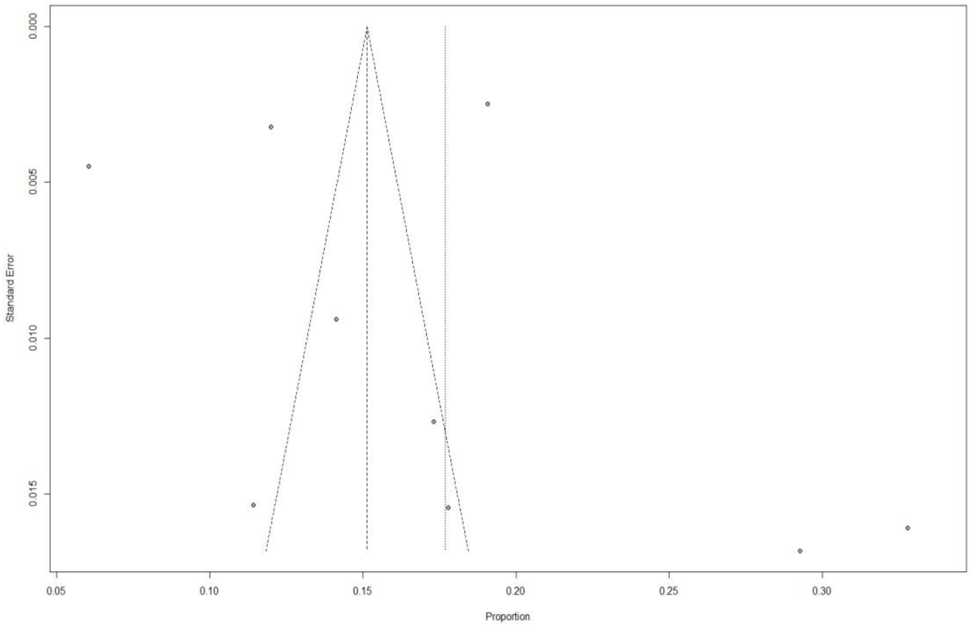

S 2.1：Forest Plot Of Chinese Mainland and Taiwan

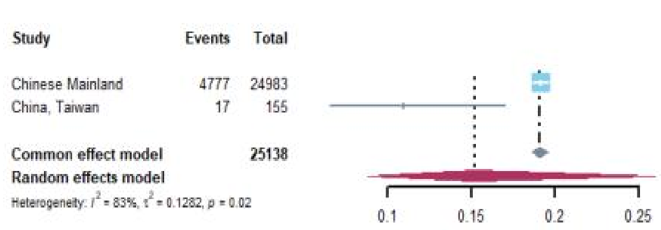

S 2.2：Sensitivity Analysis Chart Of Chinese Mainland and Taiwan

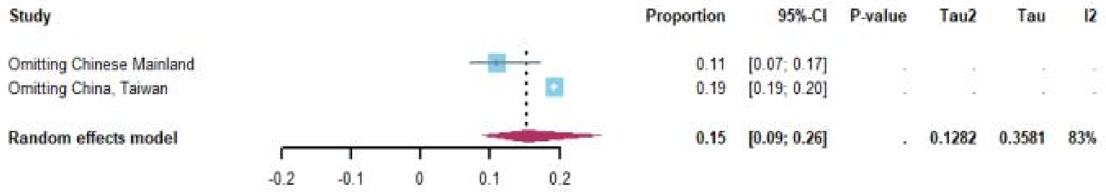

S2.3：Funnel Plot Of Chinese Mainland and Taiwan

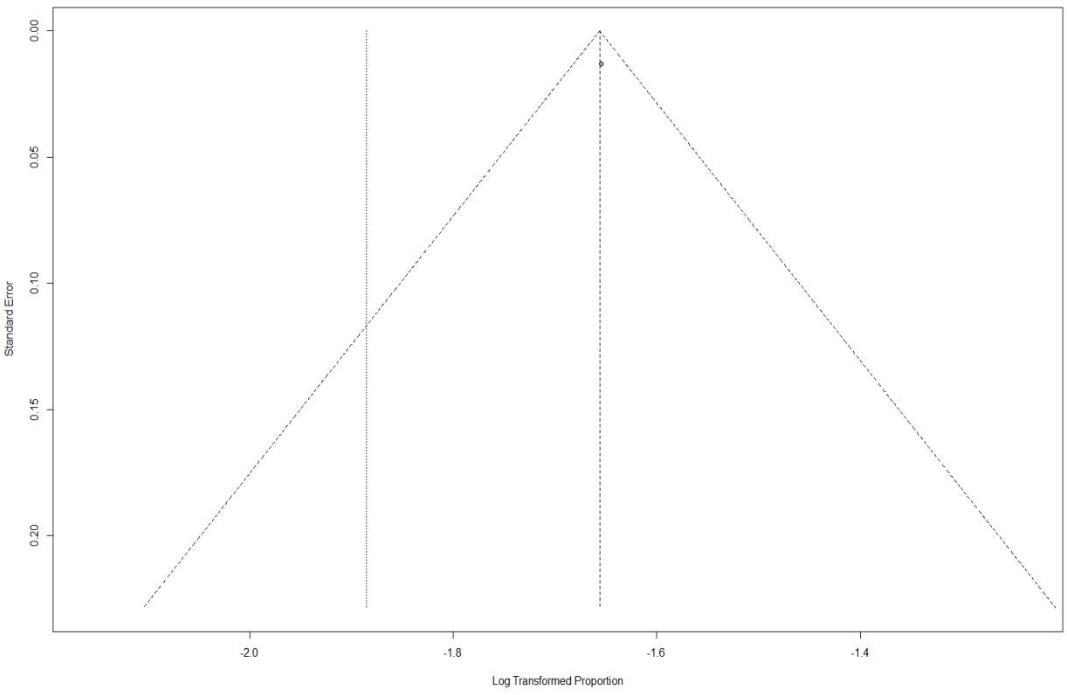

S 3.1：Forest Plot Of Developed And Developing Countries

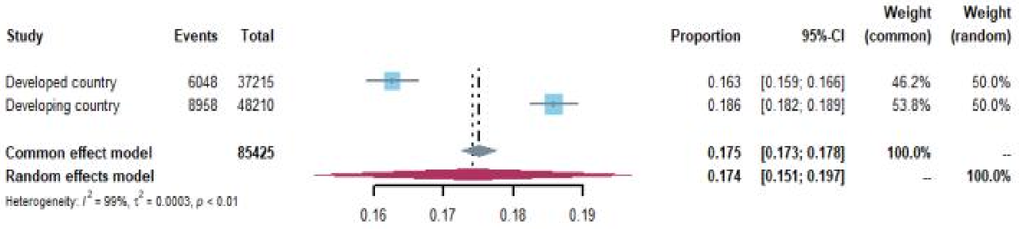

S 3.2：Sensitivity Analysis Chart Of Developed And Developing Countries

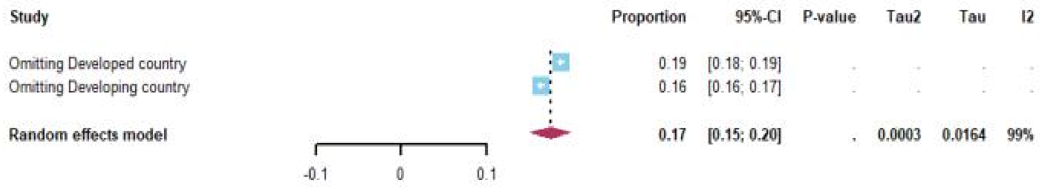

S 3.3：Funnel Plot Of Developed And Developing Countries

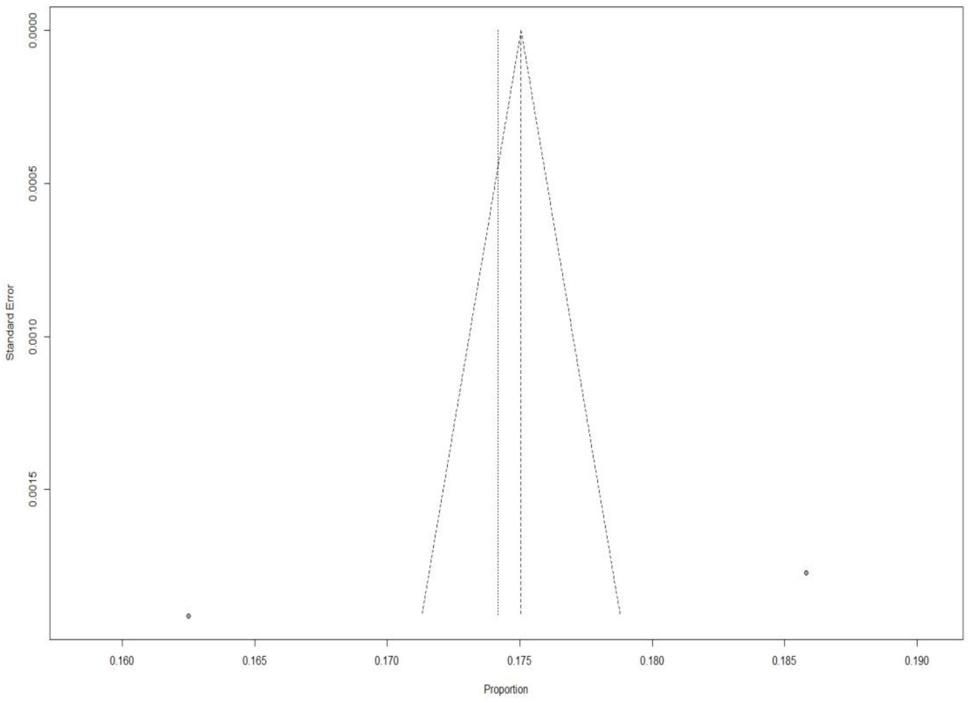

S 4.1：Forest Plot Of Countries In The Southern Hemisphere And Northern Hemisphere

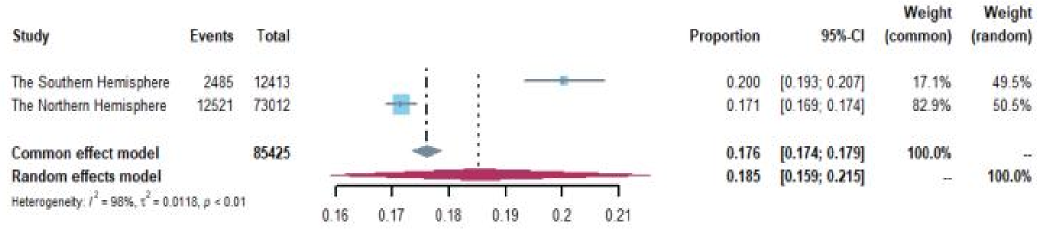

S 4.2：Sensitivity Analysis Chart Of Countries In The Southern Hemisphere And Northern Hemisphere

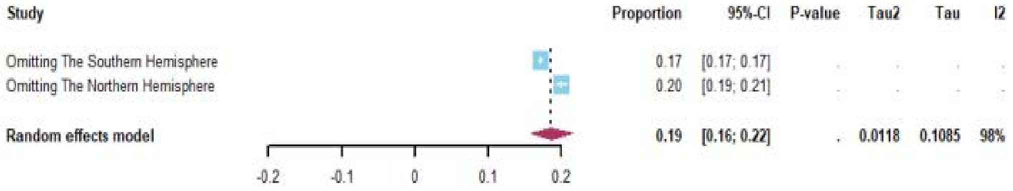

S 4.3：Funnel Plot Of Countries In The Southern Hemisphere And Northern Hemisphere

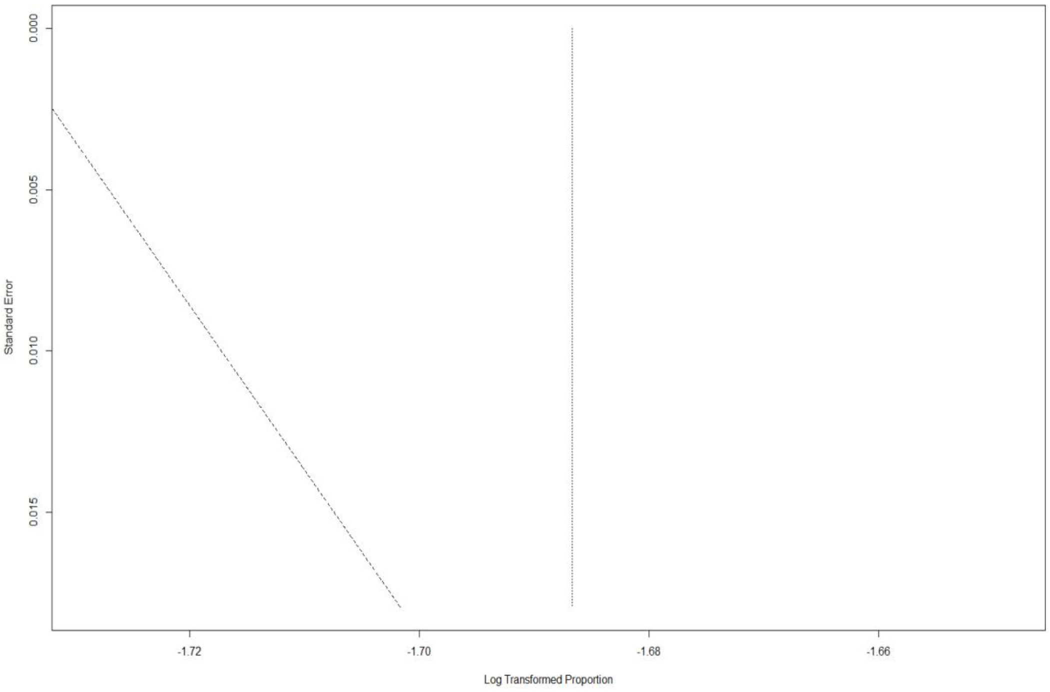

S 5.1：Forest Plot Of Different Months

Month	Proportion	95%-CI	Weight (common)	Weight (random)
N Jan,S Jul	0.234	[0.215; 0.255]	6.7%	8.3%
N Feb,S Aug	0.188	[0.168; 0.209]	6.4%	8.3%
N Mar,S Sep	0.220	[0.199; 0.242]	5.9%	8.3%
N Apr,S Oct	0.171	[0.151; 0.193]	6.1%	8.3%
N May,S Nov	0.122	[0.105; 0.141]	8.3%	8.3%
N Jun,S Dec	0.068	[0.055; 0.084]	13.4%	8.4%
N Jul,S Jan	0.069	[0.056; 0.083]	14.9%	8.4%
N Aug,S Feb	0.091	[0.077; 0.107]	12.2%	8.4%
N Sep,S Mar	0.122	[0.105; 0.141]	8.6%	8.4%
N Oct,S Apr	0.203	[0.183; 0.225]	6.1%	8.3%
N Nov,S May	0.233	[0.212; 0.256]	5.7%	8.3%
N Dec,S Jun	0.263	[0.242; 0.285]	5.7%	8.3%
	**0.143**	**[0.138; 0.148]**	**100.0%**	--
	**0.165**	**[0.126; 0.204]**	--	**100.0%**

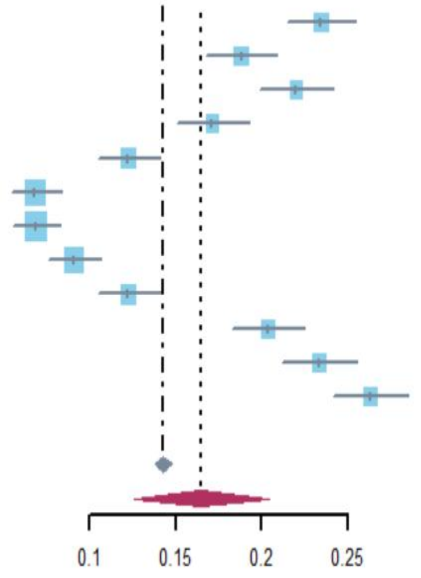

（N：the Northern Hemisphere；S：the Southern Hemisphere）

S 5.2：Sensitivity Analysis Chart Of Different Months

Month	Proportion	95%-CI	P-value	Tau2	Tau	I2
N Jan,S Jul	0.16	[0.12; 0.20]	.	0.0046	0.0678	98%
N Feb,S Aug	0.16	[0.12; 0.21]	.	0.0051	0.0711	98%
N Mar,S Sep	0.16	[0.12; 0.20]	.	0.0048	0.0692	98%
N Apr,S Oct	0.16	[0.12; 0.21]	.	0.0051	0.0715	98%
N May,S Nov	0.17	[0.13; 0.21]	.	0.0049	0.0702	98%
N Jun,S Dec	0.17	[0.14; 0.21]	.	0.0041	0.0640	98%
N Jul,S Jan	0.17	[0.14; 0.21]	.	0.0041	0.0640	98%
N Aug,S Feb	0.17	[0.13; 0.21]	.	0.0045	0.0672	98%
N Sep,S Mar	0.17	[0.13; 0.21]	.	0.0049	0.0702	98%
N Oct,S Apr	0.16	[0.12; 0.20]	.	0.0050	0.0704	98%
N Nov,S May	0.16	[0.12; 0.20]	.	0.0046	0.0679	98%
N Dec,S Jun	0.16	[0.12; 0.19]	.	0.0041	0.0639	98%
	**0.17**	**[0.13; 0.20]**	**.**	**0.0046**	**0.0682**	**98%**

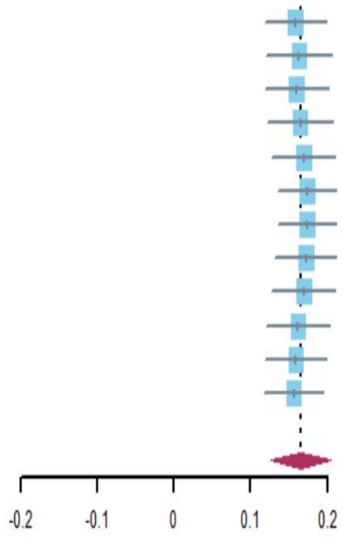

（N：the Northern Hemisphere；S：the Southern Hemisphere）

S 5.3：Funnel Plot Of Different Months

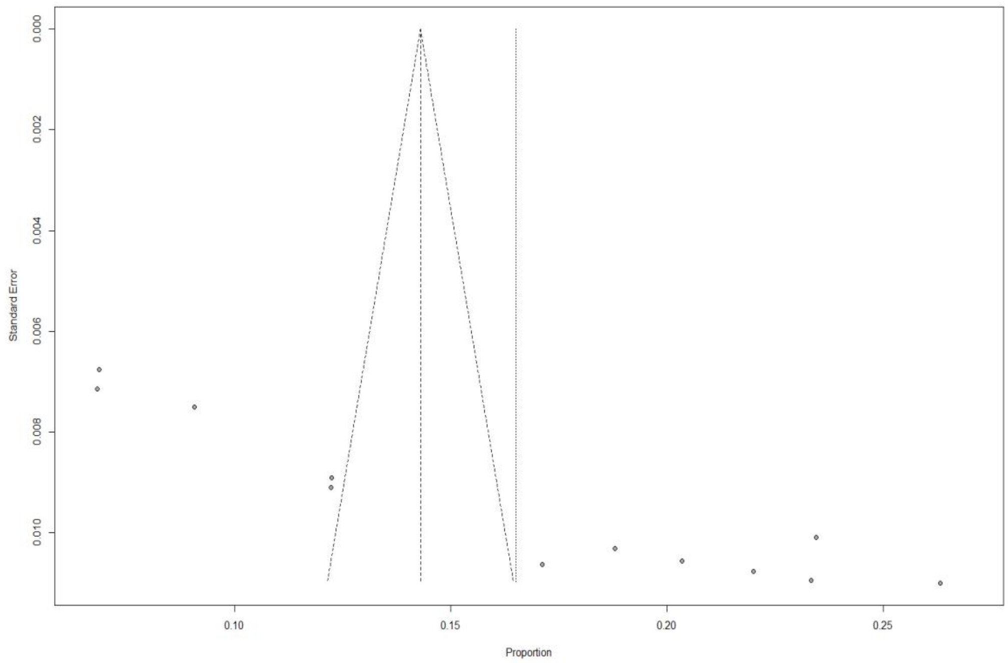

S 6.1：Forest Plot Of Different Seasons

Season
Spring
Summer
Autumn
Winter

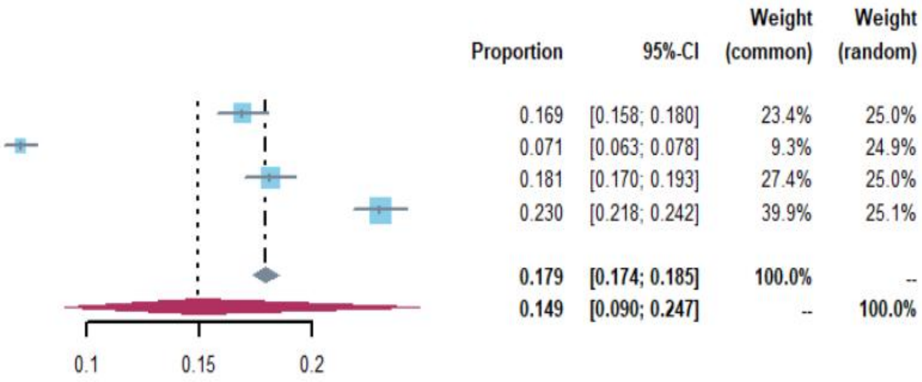

(Spring:the Northern Hemisphere March-May， the Southern Hemisphere September-November; Summer:the Northern Hemisphere June-August，the Southern Hemisphere December-February; Autumn:the Northern Hemisphere September-November，the Southern Hemisphere March-May; Winter:the Northern Hemisphere December-February，the Southern Hemisphere June-August)

S 6.2：Sensitivity Analysis Chart Of Different Seasons

Season
Spring
Summer
Autumn
Winter

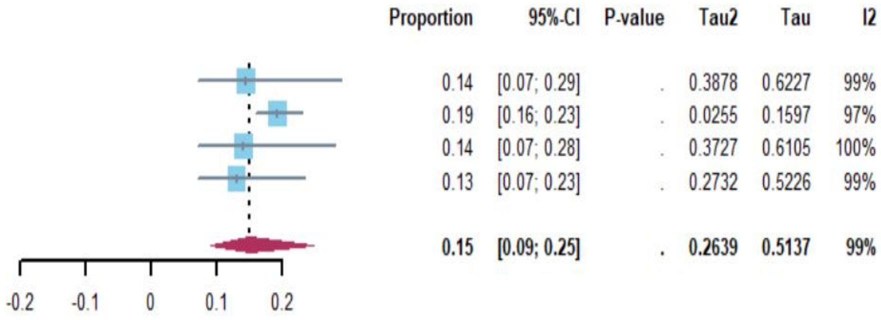

S 6.3：Funnel Plot Of Different Seasons

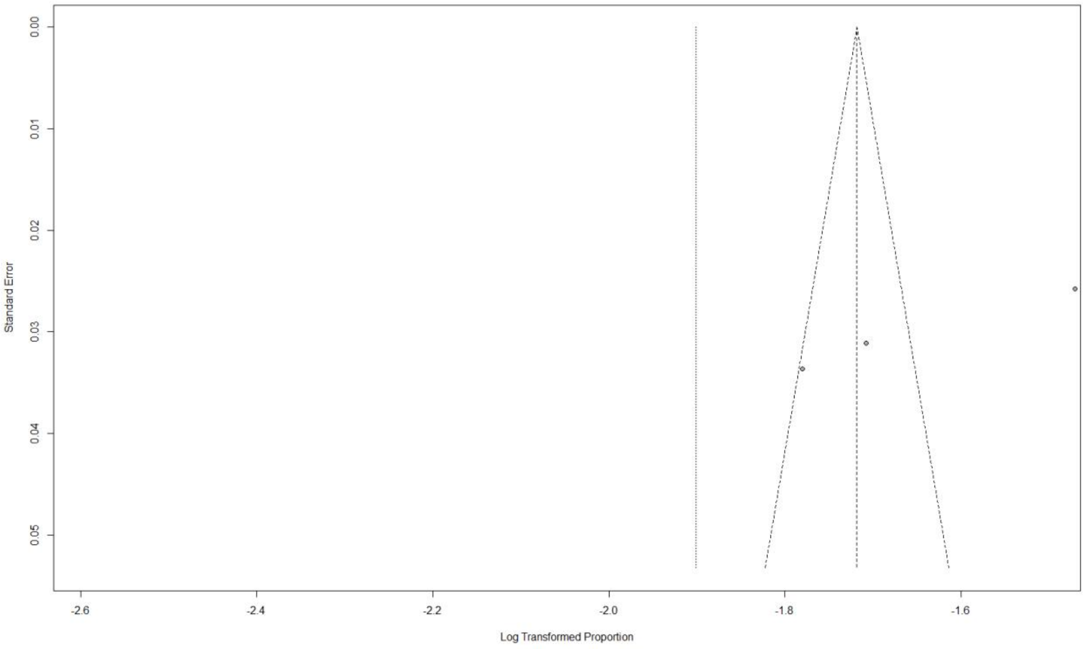

S 7.1：Forest Plot Of Warm And Cold Months

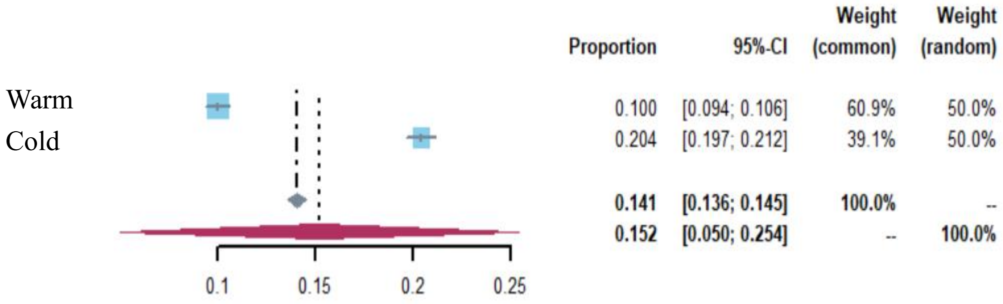

(Warm:the Northern Hemisphere April-September， the Southern Hemisphere October-March; Cold:the Northern Hemisphere October-March，the Southern Hemisphere April-September）

S 7.2：Sensitivity Analysis Chart Of Warm And Cold Months

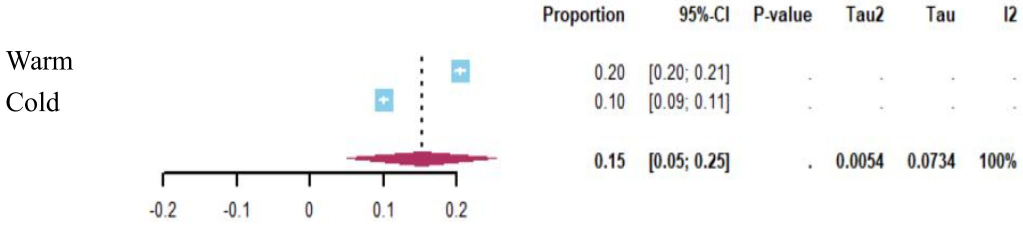

S 7.3：Funnel Plot Of Warm And Cold Months

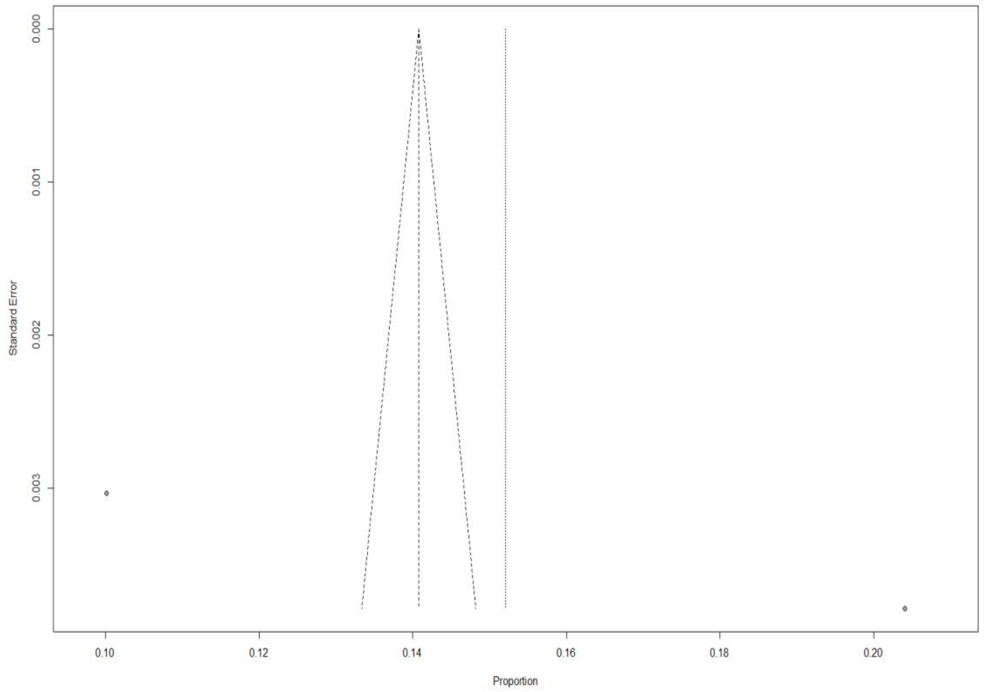

S 8.1：Forest Plot Of Different Ages（1）

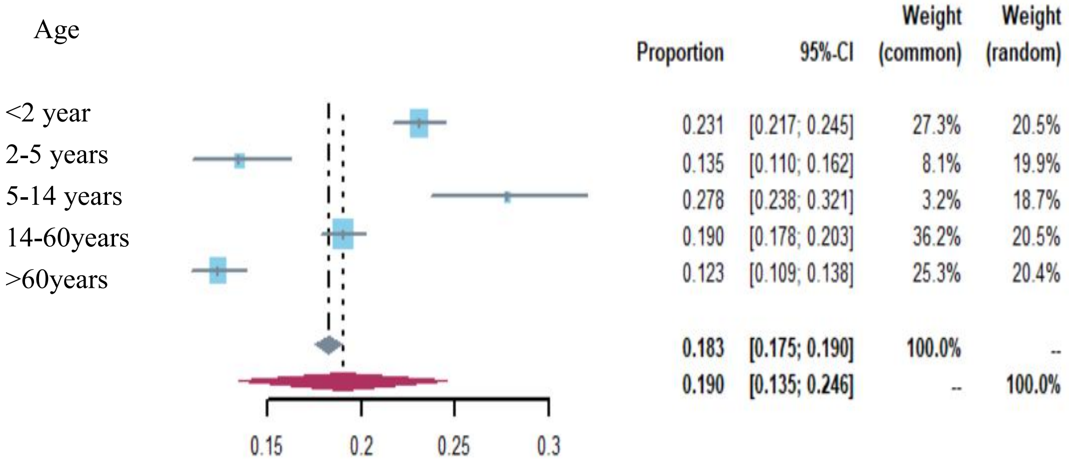

S 8.2：Sensitivity Analysis Chart Of Different Ages（1）

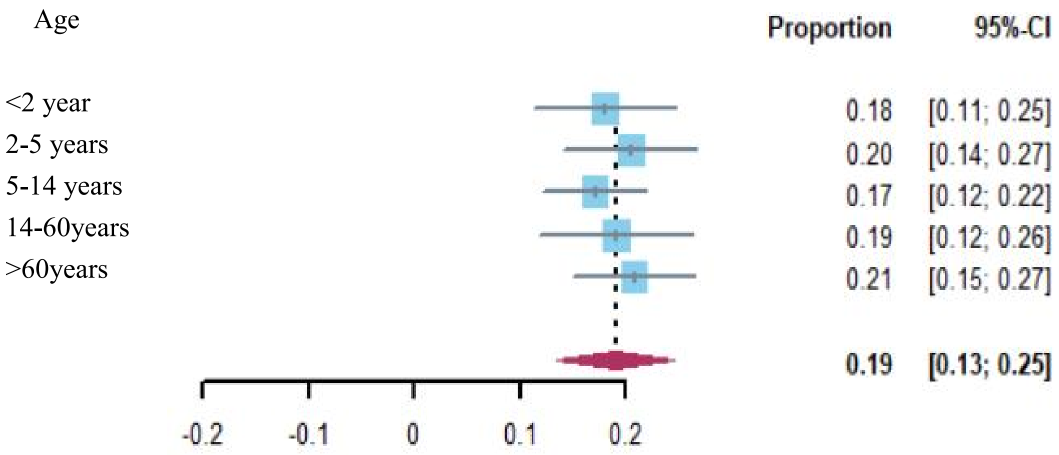

S 8.3：Funnel Plot Of Different Ages（1）

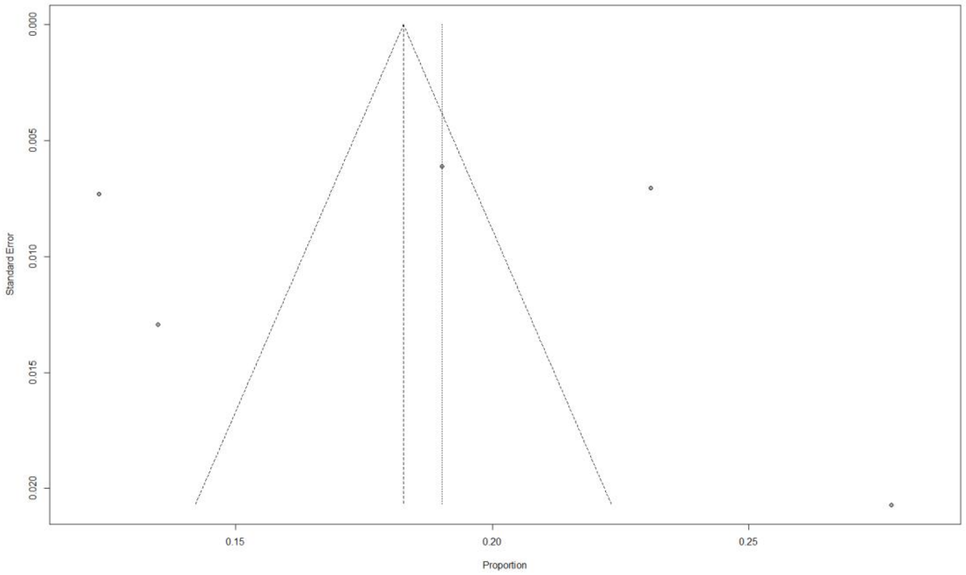

S 9.1：Forest Plot Of Different Ages（2）

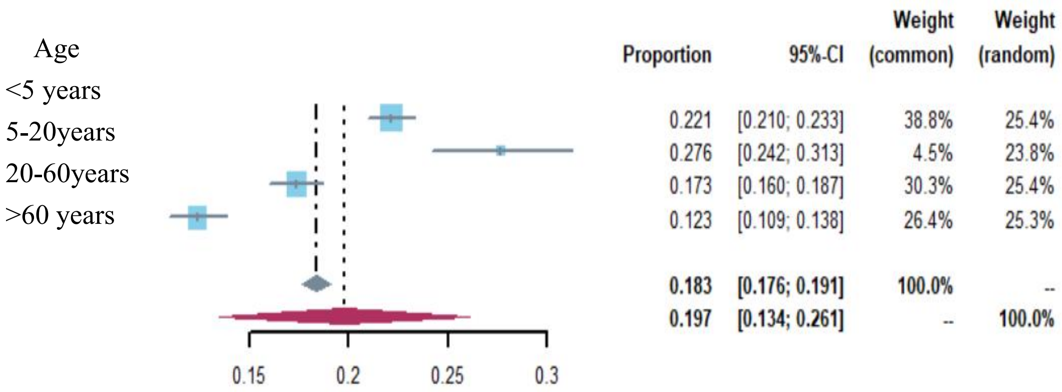

S 9.2：Sensitivity Analysis Chart Of Different Ages（2）

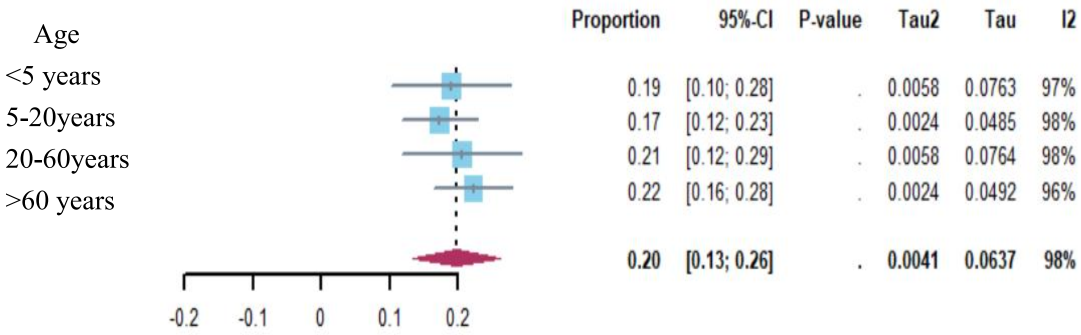

S 9.3：Funnel Plot Of Different Ages（2）

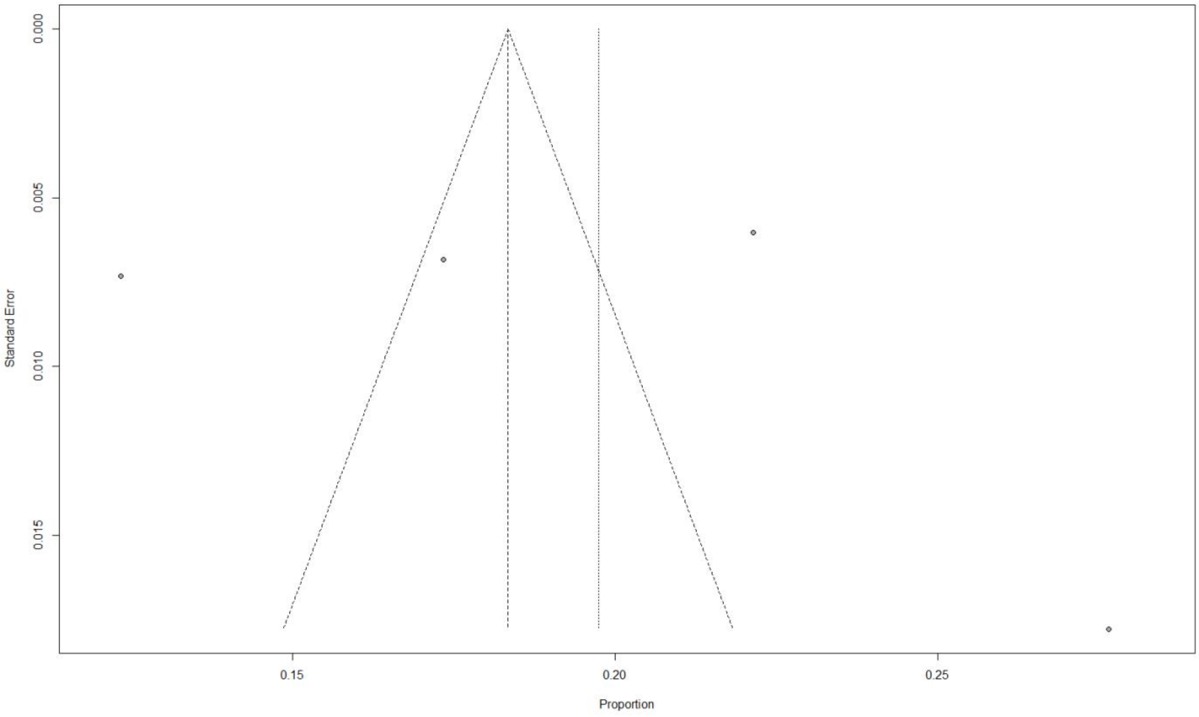

S 10.1：Forest Plot Of Sex

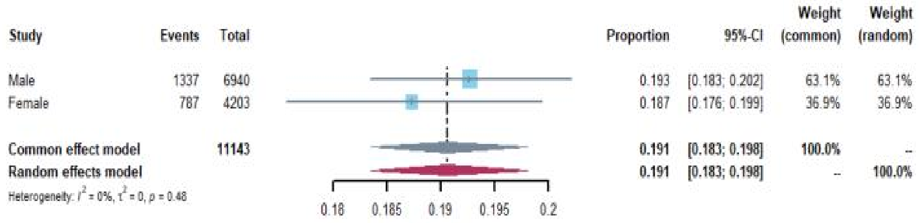

S 10.2：Sensitivity Analysis Chart Of Sex

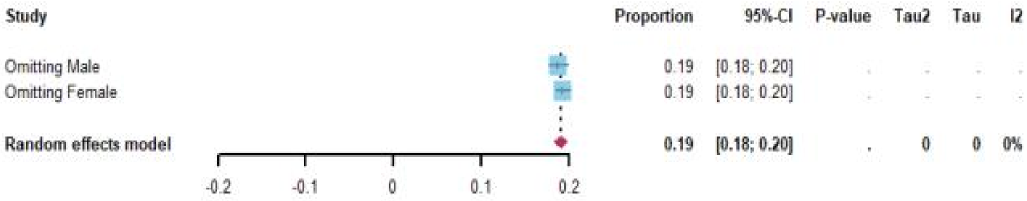

S 10.3：Funnel Plot Of Sex

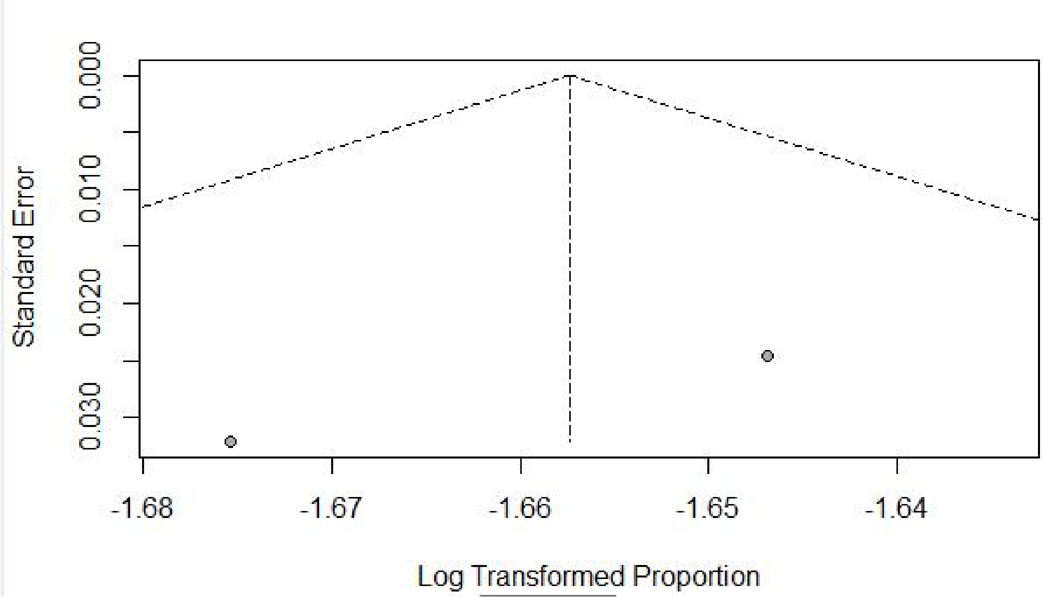

S 11.1：Sensitivity Analysis Chart Of Different Years

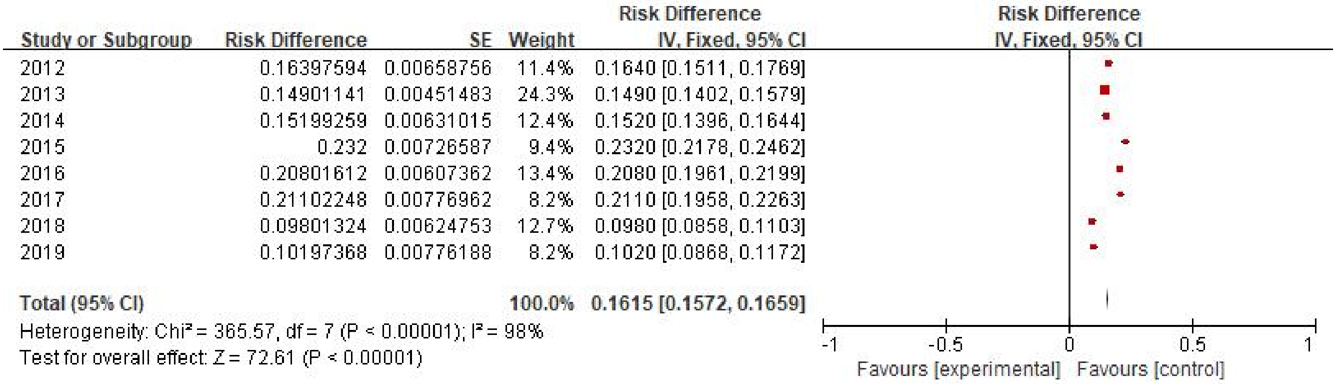

Study or Subgroup	Risk Difference	SE	Weight	Risk Difference IV, Fixed, 95% CI
2012	0.16397594	0.00658756	11.4%	0.1640 [0.1511, 0.1769]
2013	0.14901141	0.00451483	24.3%	0.1490 [0.1402, 0.1579]
2014	0.15199259	0.00631015	12.4%	0.1520 [0.1396, 0.1644]
2015	0.232	0.00726587	9.4%	0.2320 [0.2178, 0.2462]
2016	0.20801612	0.00607362	13.4%	0.2080 [0.1961, 0.2199]
2017	0.21102248	0.00776962	8.2%	0.2110 [0.1958, 0.2263]
2018	0.09801324	0.00624753	12.7%	0.0980 [0.0858, 0.1103]
2019	0.10197368	0.00776188	8.2%	0.1020 [0.0868, 0.1172]
**Total (95% CI)**			**100.0%**	**0.1615 [0.1572, 0.1659]**

Heterogeneity: Chi² = 365.57, df = 7 (P < 0.00001); I² = 98%

Test for overall effect: Z = 72.61 (P < 0.00001)

S 11.2：Funnel Plot Of Different Years

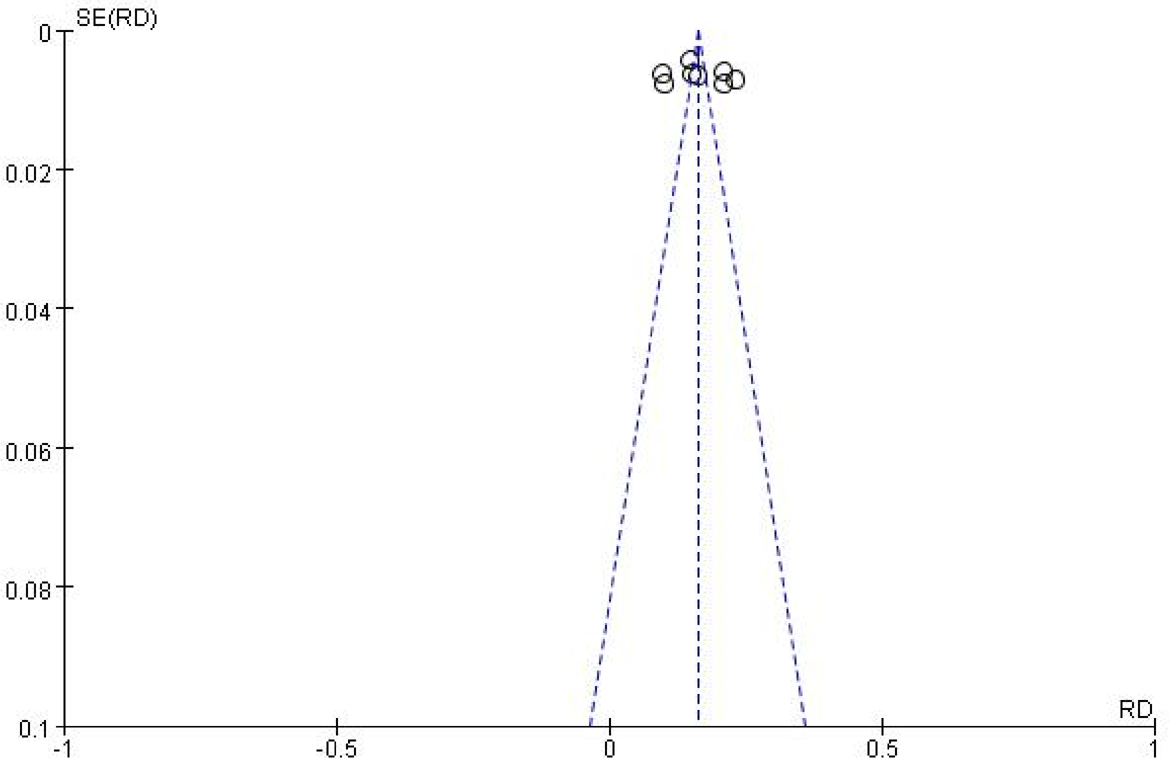

S 12: the sample size calculation of each sub-group

Characteristics	Cases of studies	Pooled prevalence(%) (95% CI)	Included sample size	required sample size
Age groups (1)	2082	19.0(14.3-23.7)	10598	262—409
Age groups (2)	2003	19.7(14.5-24.9)	10168	345—391
Sex	2124	19.1(18.3-19.8）	11120	260—407
Years	4695	16.0(15.6-16.4)	29344	323—504
Month	2929	16.5(12.5-20.5)	17752	311—486
Season	3068	16.3(8.8-23.7)	18822	316—493
Temperature	3296	15.2(5.0-25.4)	21684	343—536
Counties	15089	19.0(16.7-21.4)	79416	262—409
